# Engineered oncolytic bacteria HCS1 exerts high immune stimulation and safety profiles for cancer therapy

**DOI:** 10.7150/thno.87340

**Published:** 2023-10-16

**Authors:** Yujie Sun, Yanxia Guo, Xiaoqing Liu, Jinling Liu, Honglai Sun, Zhongying Li, Min Wen, Sheng-Nan Jiang, Wenzhi Tan, Jin Hai Zheng

**Affiliations:** 1 School of Biomedical Science, Hunan University, Changsha 410082, China.; 2 College of Biology, Hunan University, Changsha 410082, China.; 3 Department of Neurosurgery, Guangzhou First People′s Hospital, Guangzhou 510180, China.; 4 Department of Nuclear Medicine, the Second Affiliated Hospital of Guangzhou Medical University, Guangzhou, 510260, China.; 5 School of Food Science and Bioengineering, Changsha University of Science & Technology, Changsha, Hunan 410114, China.

**Keywords:** bacterial-mediated cancer therapy, *Salmonella typhimurium*, VNP20009, systemic safety, clinical translation

## Abstract

**Background and rationale:** Attenuated *Salmonella typhimurium* VNP20009 has been used to treat tumor-bearing mice and entered phase I clinical trials. However, its mild anticancer effect in clinical trials may be related to insufficient bacterial colonization and notable adverse effects with increasing dosages. Guanosine 5′-diphosphate-3′-diphosphate (ppGpp) synthesis-deficient *Salmonella* is an attenuated strain with good biosafety and anticancer efficacy that has been widely investigated in various solid cancers in preclinical studies. Integration of the advantages of these two strains may provide a new solution for oncolytic bacterial therapy.

**Methods:** We incorporated the features of ΔppGpp into VNP20009 and obtained the HCS1 strain by deleting *relA* and *spoT*, and then assessed its cytotoxicity *in vitro* and antitumor activities *in vivo*.

**Results:** *In vitro* experiments revealed that the invasiveness and cytotoxicity of HCS1 to cancer cells were significantly lower than those of the VNP20009. Additionally, tumor-bearing mice showed robust cancer suppression when treated with different doses of HCS1 intravenously, and the survival time and cured mice were dramatically increased. Furthermore, HCS1 can increase the levels of pro-inflammatory cytokines in tumor tissues and relieve the immunosuppression in the tumor microenvironments. It can also recruit abundant immune cells into tumor tissues, thereby increasing immune activation responses.

**Conclusion:** The newly engineered *Salmonella* HCS1 strain manifests high prospects for cancer therapeutics and is a promising option for future clinical cancer immunotherapy.

## Introduction

Cancer is a devastating disease that poses a serious threat to human health. Although traditional cancer therapies, such as surgery, chemotherapy, radiation therapy, and immunotherapy, remain the standard therapeutic modalities for many cancers, they have several limitations, including toxicity to normal tissues, poor tissue penetration, and multi-drug resistance 1. Consequently, alternative treatments such as gene therapy and biotherapy have emerged as potential ways to improve the efficacy of cancer therapy 2,3. Bacteria-mediated cancer therapy (BMCT) was first introduced in 1891 when Dr. William Coley injected a mixture of heat-inactivated* Serattia marcescence* and *Streptococcus pyogenes* into patients with inoperable cancers 4. Tumors generally exhibit characteristics such as hypoxia, immunosuppression, and a nutrient-rich environment, which favor the colonization and reproduction of bacteria. Facultative or obligate anaerobic bacteria, such as *Listeria monocytogenes* 5,6, *Bifidobacterium* 7,8, *Escherichia coli* 9,10, and *Salmonella* 11-13, exert intrinsic tumor-targeting and oncolytic activities. Moreover, bacteria, acting as drug-delivery vehicles and immune modulators in tumor biotherapy, have attracted attention with the advancement of biotechnology and immunology owing to their unique properties, including tumor targeting and localization and therapeutic agent production 14-17.

VNP20009 is obtained by deleting the *msbB* and *purI* genes from *Salmonella typhimurium* 14028s and has been used to treat mice bearing various cancer types 18-20. However, in phase I clinical studies, although detectable bacterial colonization was noted in all treated patients, no significant antitumor effect was observed even at the highest tolerated dose 21,22. Thus, further exploration is needed for achieving the decent antitumor effect of VNP20009 for clinical translation.

Guanosine 5′-diphosphate-3′-diphosphate (ppGpp) plays a vital role in regulating bacterial growth and cell size 23. RelA is a monofunctional ppGpp synthetase enzyme that is activated in response to amino acid starvation 24. Another enzyme, SpoT, possesses strong ppGpp hydrolase and weak synthetase activity 25. Deletion of both *relA* and *spoT* genes results in a ppGpp-deficient strain known as ΔppGpp, which was reported to increase the half-lethal dose in mice by 100,000-1,000,000-fold compared with that by the parental *Salmonella* strain 14028s 26. *In vitro* studies have shown that ΔppGpp has reduced invasiveness toward tumor cells and lower virulence than VNP20009, and it can achieve better therapeutic outcomes at equivalent doses 27. Furthermore, ΔppGpp has been used alone or in combination with chemotherapy or radiotherapy for enhanced cancer treatments 28,29 and can serve as a protein expression vector or BMCT 30-32.

Therefore, we aimed to incorporate the advantages of the ΔppGpp into VNP20009 to improve safety and therapeutic effect. By using suicide plasmid-mediated homologous recombination, we deleted the *relA* and *spoT* genes in VNP20009 to obtain a new strain and termed as HCS1. Results showed that metabolic pathways had great changed in HCS1 compared with VNP20009, and HCS1 had a substantially lower invasion of tumor cells *in vitro* and produced extremely low cytotoxicity even at a high dose by intracellular bacteria counts and lactate dehydrogenase (LDH) assays. Moreover, compared with VNP20009, HCS1 exerted better therapeutic effects and longer animal survival in subcutaneous xenografted tumor-bearing mice. Systemic toxicity was confirmed by clinical chemistry parameter measurement and histological pathology examination, which revealed better safety profiles than the VNP20009. Tumor microenvironments (TMEs) analysis indicated abundant immune cell infiltration and robust immune activation as checked by immunofluorescence staining and RNA-sequencing. Taken together, these findings suggest HCS1 is a promising bacterial strain for clinical application.

## Materials and Methods

### Bacterial strains

The *S. typhimurium* strain VNP20009 was obtained as previously described 33. To obtain a therapeutic payload, *clyA* was cloned into the pBAD vector (pBAD-ClyA) 34. This plasmid was then transformed into both the VNP20009 and HCS1 strains, resulting in the production of VNP20009^ClyA^ and HCS1^ClyA^, respectively.

### Construction of HCS1

Using a suicide plasmid-mediated homologous recombination method 35,36, we knocked out *relA* and *spoT* from the VNP20009 genome to create the HCS1 strain. Specifically, we first cloned the upstream and downstream sequences of *relA* into the suicide vector pDM4 plasmid, and the recombinant pDM4 vector was then transformed into a conjugative *E. coli* strain SM10 λpir. The mutant allele was transferred into VNP20009 through biparental mating, and the vector containing the deletion allele was integrated into the chromosome of *Salmonella* through homologous recombination. The resulting merodiploid was selected using the chloramphenicol agar plates. A second homologous recombination was then spontaneously performed, and the suicide marker *sacB* was used to counterselect and isolate colonies that had lost the plasmid backbone. Sucrose-resistant colonies were checked using colony PCR, and the deletion of *relA* was further confirmed using DNA sequencing. The same process was repeated to knock out* spoT*. The strains and plasmids used are presented in Table S1, and the primers used for amplification are listed in Table S2.

### Cell culture

The MC38 and CT26 cells were cultured in Dulbecco's Modified Eagle's Medium (DMEM) supplemented with 10% heated-inactivated fetal bovine serum (FBS), 100 U/mL penicillin, and 100 μg/mL streptomycin (Gibco). These cells were maintained in a humidified atmosphere containing 5% CO_2_ at 37℃.

### Bacterial growth curve

The *Salmonella* strains 14028s, VNP20009, and HCS1 were cultured in LB medium. Single colonies were cultured overnight and harvested via centrifugation at 6,000 rpm (5424R, Eppendorf) for 2 min, followed by washing and diluting with PBS (pH 7.4). Subsequently, 3×10^8^ CFU were inoculated into 30 mL LB medium and incubated with 200 rpm shaking at 37 ℃. The OD_600_ of each strain was measured at 0, 2, 4, 6, 8, 10, 12, and 24 h using a UV spectrophotometer (Shimadzu, UVmini-1280).

### Bacterial invasion assay

The MC38 and CT26 cells were seeded at a density of 1×10^5^ cells per well in 24-well plates and incubated overnight in an incubator at 37 ℃ and 5% CO_2_. The culture media were removed, and the cells were washed twice with Dulbecco's PBS (DPBS). DMEM (basic medium without additional supplement, 500 μL per well) was added for further culture. VNP20009 and HCS1 were activated in fresh LB media (1:100) for 4 h after overnight culture. The bacteria were collected and washed twice with PBS, and the bacterial concentration was quantified by measuring OD_600_ with a UV spectrophotometer (1 OD_600_ = 8×10^8^ CFU/mL). VNP20009 and HCS1 were used for cell infection with MOIs at 10 and 100, respectively. After 1 h of bacterial infection, the medium was removed, and the cells were washed twice with DPBS. To quantify the intracellular bacteria, DMEM containing 150 μg/mL gentamycin was added for culturing for an additional 30 min. The medium was then removed, and the cells were washed three times with DPBS. Subsequently, cell lysates were prepared with 0.01% Triton X-100 in PBS, diluted, and spotted on LB plates (dilution factor: 10^0^-10^3^). The plates were incubated overnight at 37 ℃, and the colonies were counted and calculated the next day based on the dilution factors.

### Crystalline violet staining

The MC38 and CT26 cells were seeded in six-well plates at a density of 3×10^5^ cells per well and incubated overnight. The next day, the culture medium was removed, and the cells were washed twice with DPBS. Thereafter, 2 mL DMEM containing 10% FBS was added. VNP20009 and HCS1 were used to infect the cells at MOI 1000. After 6 h of co-culture, the supernatants were removed, and the cells were washed twice with DPBS. Complete DMEM medium supplemented with 150 μg/mL gentamycin was added to continue the culture. After 24 h, the cells were washed twice with DPBS, fixed with 4% PFA at room temperature for 10 min, and then washed twice with DPBS. The cells were stained with 1 mL of 0.1% crystal violet dye and incubated for another 30 min, followed by washing twice with DPBS, and the photographs were obtained.

### Lactate dehydrogenase assay

The MC38 and CT26 cells were seeded in 24-well plates at a density of 1×10^4^ cells per well and incubated overnight. VNP20009 and HCS1 were added at MOI 100 for 6 h, and 150 μg/mL gentamycin was added for another 12 h of culture. The cell culture supernatants were examined using an LDH assay (CytoTox 96 Non-Radioactive Cytotoxicity Assay) following the manufacturer's instructions. The absorbance of the sample at 490 nm was measured using a microplate reader (Molecular Devices, SpectraMax M2/M2e, United States), and the LDH release was calculated.

### Mice models

Six-week-old female C57BL/6 or BALB/c mice were purchased from Gempharmatech (Jiangsu, China). All mice were raised in a specific pathogen-free environment with a standard 12 h light/dark diurnal restriction. Sterilized water and food were given *ad libitum*. Mice were shaved and placed under anesthesia in an induction box containing 2.5% isoflurane. The MC38 and CT26 cells were subcutaneously implanted into the right flank of the mice at a density of 1×10^6^ each. When the tumors reached a size of 100-120 mm^3^, different treatments were applied. On the day defined as 0-day post-inoculation (dpi), the mice were intravenously injected with 100 μL of PBS containing either 1×10^7^ CFU of VNP20009 or 1×10^7^, 5×10^7^, or 1×10^8^ CFU of HCS1. The tumor sizes and weight of mice were measured and recorded every 3 days until the tumor volumes reached ≥1,500 mm^3^. The tumors were measured with a caliper, and the tumor volume was calculated using the following formula: (L × W × H)/2, where L, W, and H are the length, width, and height, respectively, of the tumor in millimeters. Mice with tumor volumes ≥1500 mm^3^ were euthanized following the guidelines of the Animal Research Committee at Hunan University, China.

### Bacterial distribution *in vivo*

Tumor-bearing mice were intravenously injected with bacteria (harboring empty vector with ampicillin resistance) in 100 μL of PBS. The spleens, livers, and tumors were collected at 3 and 7 dpi and homogenized, and the collected homogenates were serially diluted and plated onto LB agar plates with ampicillin selection, and viable bacteria was counted. VNP20009 and HCS1 strain were transduced with bacterial luciferase operon (*lux*) to obtain VNP20009^lux^ and HCS1^lux^, respectively. Then the optical bioluminescence images were performed with an *in vivo* imaging system (IVIS SpectrumCT, Perkin Elmer). Data statistical analysis was performed using the Mann-Whitney *U* test.

### FACS assay

Immune cells were harvested from the tumor-draining lymph nodes of MC38-bearing mice and disrupted mechanically. The obtained cells were then passed through a 40 μm cell strainer to generate single-cell suspensions. The cells were incubated with anti-Gr-1-PE (12-5931, eBioscience), anti-F4/80-PE (12-3110, eBioscience), anti-CD11c-PE (12-0114, eBioscience), and anti-CD11b-FITC (11-0112, eBioscience) antibodies following the manufacturer's instructions. Subsequently, 20,000 events were recorded using BD (FACSCelesta), and the acquired data were analyzed using the FlowJo V10 software (Tree Star). The Gr-1^+^ cells were identified as neutrophils, whereas CD11b^+^ F4/80^+^ and CD11b^+^ CD11c^+^ cells were identified as macrophages and dendritic cells, respectively.

### H&E and immunofluorescence staining

After euthanizing the mice with a CO_2_ chamber, their organs (hearts, lungs, livers, spleens, and kidneys) and subcutaneous tumors were collected and fixed in 4% paraformaldehyde. The organs and tumors were embedded in an optimal cutting temperature compound or paraffin, and then 6 μm thick sections were cut using a freezing microtome (LEICA CM1950, Germany) or HistoCore Biocut (LEICA 14051756235, Germany) and mounted onto slides. H&E staining was performed following standard protocols, and the sections were photographed using a Panthera upright compound microscope (Panthera S, Motic). For immunofluorescence staining, contiguous sections were stained overnight at 4 ℃ with primary anti-neutrophil (SC-71674, Santa Cruz), anti-F4/80 (MCA497GA, AbD Serotec), anti-CD11c-PE (12-0114, eBioscience), anti-Ki67 (27309-1-Ap), anti-*Salmonella* (Ab35156, Abcam) antibodies. The sections were then washed and stained with secondary antibodies, donkey anti-rat 594 (A-21209, Invitrogen), donkey anti-rabbit 488 (A-21206, Invitrogen) or donkey anti-rabbit 555 (A-31572, Invitrogen). The TUNEL assay was performed with standard protocols (A113-01, Vazyme). The sections were photographed using laser scanning confocal microscopy (LSM 980, Zeiss).

### ELISA

For *in vitro* stimulation of macrophages, RAW264.7 cells were incubated with bacteria for 6 and 12 h. After incubation, the supernatants were collected, and levels of TNF-α and IL-1β were measured. For analyzing cytokine activation in tumors, tumor tissues were collected and lysed using RIPA buffer containing proteinase inhibitor at the indicated times. The supernatants were collected for determining the cytokine levels (TNF-α, IL-1β, and IL-10), using ELISA kits as per the manufacturer's protocol (eBioscience).

### Clinical chemistry parameter analysis

At 7 dpi, serum was collected from the mice, and clinical chemistry parameters, including aspartate aminotransferase (AST) and alanine aminotransferase (ALT) levels, were analyzed using an automatic biochemical analysis machine (Mindray BS-860, China).

### Western blot analysis

VNP20009^ClyA^ and HCS1^ClyA^ were first cultured in LB medium with ampicillin overnight, and the fresh culture was then grown at a ratio of 1:100 until the OD_600_ reached 0.6-0.8. The bacteria were then divided equally and with or without 0.2% L-arabinose induction for another 5 h. Subsequently, 5×10^7^ CFU of bacteria were collected and denatured for Western blot with a primary antibody, followed by a horseradish peroxidase-conjugated secondary antibody.

For Western blot analysis of tumor tissues, the tissues were homogenized and lysed in RIPA buffer supplemented with protease inhibitor cocktail at a 1:100 dilution, followed by incubation on ice for approximately 30 min. The protein concentration was determined using a NanoDrop (Thermofisher). Approximately 100 µg of total protein was used for Western blot following standard conditions with primary antibodies (diluted at 1:1000) against poly ADP-ribose polymerase (PARP; #9232S, Cell Signaling Technology), Caspase3 (#9662S, Cell Signaling Technology), and β-actin (#SC-47778, Santa Cruz). The detection was performed using an ECL plus Western blot detection system (Sagecreation Mini chemi 910, China), and the intensity was quantitatively analyzed using the ImageJ software.

### RNA sequencing and analysis

Total RNA was extracted from frozen mouse tumor tissues using TRIzol reagent (Invitrogen, CA, USA), and its purity and quantification were assessed using a NanoDrop 2000 spectrophotometer (Thermo Scientific, USA). RNA integrity was evaluated using an Agilent 2100 Bioanalyzer (Agilent Technologies, Santa Clara, CA, USA). VAHTS Universal V6 RNA-seq library prep kit was used to construct libraries according to the manufacturer's instructions. Transcriptome sequencing and analysis were conducted by OE Biotech Co., Ltd. (Shanghai, China). The libraries were sequenced on an Illumina Novaseq 6000 platform, and 150 bp paired-end reads were generated. Raw reads in fastq format were initially processed using fastp, and low-quality reads were removed to obtain clean reads, which were retained for subsequent analyses. The clean reads were aligned to the reference genome using HISAT. The FPKM for each gene was calculated, and the read counts of each gene were obtained using HTSeq-count. Differential expression analysis was performed using DESeq2 with Q value < 0.05 and foldchange > 2 set as thresholds for significant differences in gene expression. R (v 3.2.0) was used to analyze Gene Ontology (GO) terms and evaluate functional enrichment, including the biological processes, molecular functions, and cellular components.

### Metabolomics analysis by LC-MS/MS

Bacterial pellets were collected at 4 h after fresh culture, and shipped on dry ice. The metabolomic data analysis was performed by Shanghai Luming biological technology co., LTD (Shanghai, China). An ACQUITY UPLC I-Class plus (Waters Co., Milford, MA) fitted with Q-Exactive mass spectrometer equipped with heated electrospray ionization (ESI) source (Thermo Fisher Scientific, Waltham, MA) was used to analyze the metabolic profiling in both ESI positive and ESI negative ion modes. The original LC-MS data were processed by software Progenesis QI V2.3 (Nonlinear, Dynamics, Newcastle, UK) for baseline filtering, peak identification, integral, retention time correction, peak alignment, and normalization. A two-tailed Student's *t*-test was further used to verify whether the metabolites of difference between groups were significant. Differential metabolites were selected with VIP values greater than 1.0 and *P*-values less than 0.05.

### Statistical analyses

Statistical analysis was performed using GraphPad Prism 9.0 software (San Diego, CA). The Mann-Whitney *U* test was used to determine the statistical significance of differences in tumor growth, cytokine expression, and changes in clinical chemical parameters between the control and treatment groups. Statistical significance was set at *p* < 0.05. Survival analysis was performed using the Kaplan-Meier method and log-rank test. All data are expressed as mean ± SEM.

## Results

### *In vitro* characterization of engineered HCS1 strain

**Figure 1:**
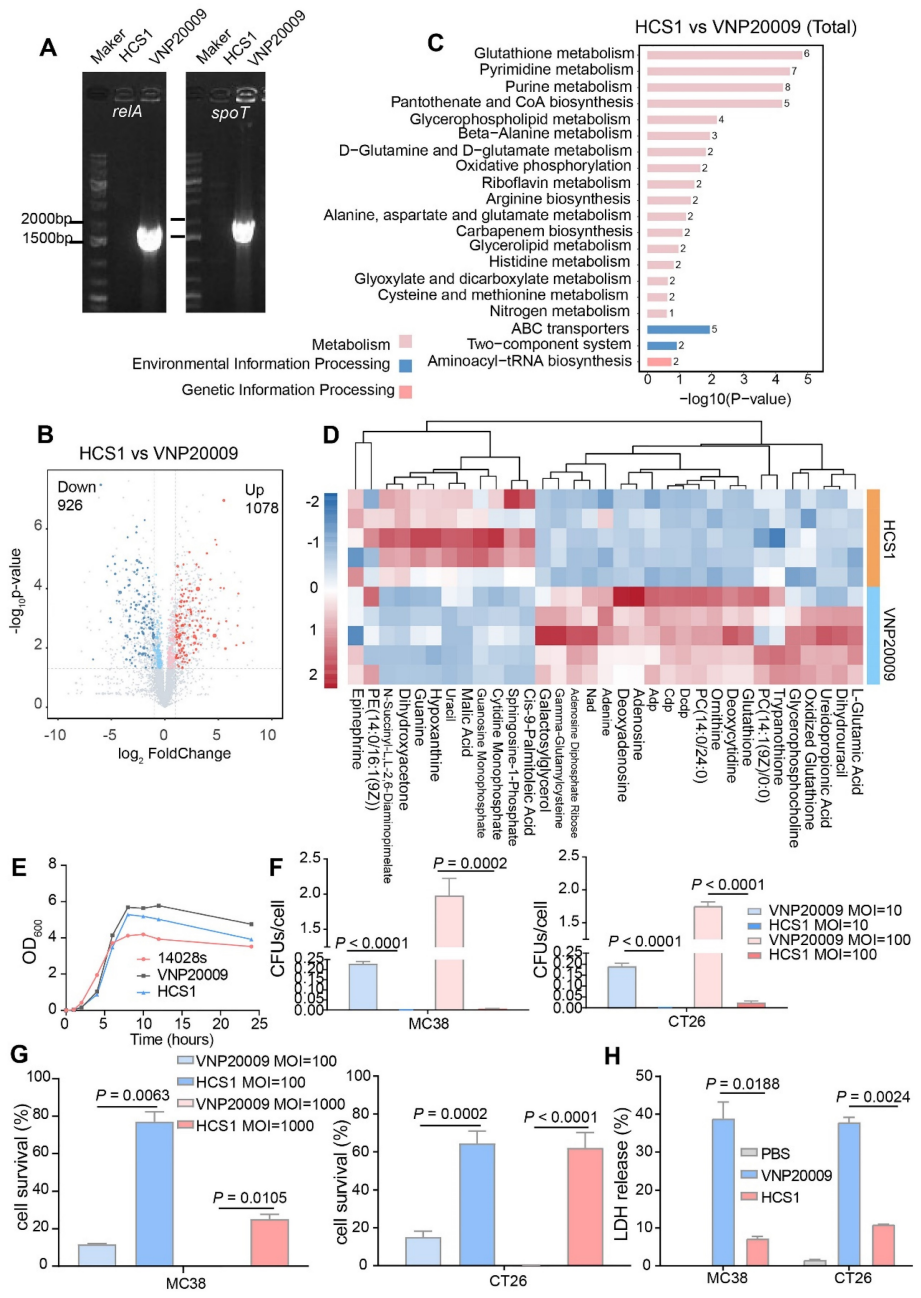
**Bacteria engineering and characterization *in vitro.*** (A) Confirmation of the deletion of *relA* and *spoT* genes in HCS1. (B-D) Metabolomics analysis of HCS1 and VNP20009 *in vitro*, presented with total metabolic changes (B), KEGG enriched metabolic pathways (C), and heat map showing the differed metabolites (D), n = 5. (E) Growth curves of the strains 14028s, VNP20009, and HCS1. (F) Number of bacteria invading cells under different infection conditions. (G) Cell viability after co-culture with bacteria at different MOI. (H) LDH released from the MC38 and CT26 cells after co-culture with HCS1 and VNP20009. Data are presented as mean ± SEM.

Firstly, the absence of *relA* and *spoT* genes in HCS1 was confirmed by PCR with specific primers (Figure 1A), and the engineered HCS1 manifested significant alterations in metabolic pathways (Figure 1B-D). The growth of the mutant was measured to explore whether the population growth of HCS1 had been affected, and growth curves were plotted. The proliferation of HCS1 was not considerably different between VNP20009 and 14028s (Figure 1E), indicating that the deficiency of ppGpp did not affect bacterial growth. Invasion ability is a crucial factor that determines bacterial cytotoxicity. The invasion ability of VNP20009 and HCS1 were measured by co-culturing with MC38 and CT26 cells at different infection coefficients (MOI 10 or MOI 100), and the cells were lysed for bacterial quantification. The results showed that the cell invasion of HCS1 with gene deletion of *relA* and *spoT* was significantly lower than that of VNP20009 both in MC38 (*p* < 0.0001 in MOI 10 and 100 groups) and CT26 (*p* < 0.0001 in MOI 10 and *p* = 0.0002 in MOI 100; Figure 1F).

To investigate the cytotoxicity of the two bacteria *in vitro*, the two strains were co-cultured with MC38 and CT26, followed by crystal violet staining (Figure S1). To further analyze cell survival *in vitro*, Trypan blue was used to differentiate the live and dead cells. The results indicated that the number of surviving cells in the VNP20009-infected group was substantially reduced, whereas no significant difference was found between the HCS1-infected and PBS-treated control groups (Figure 1G; *p* = 0.0002 and *p* < 0.0001 for CT26 cells treated with MOI 100 and 1000, respectively, and *p* = 0.0063 and *p* = 0.0105 for MC38 cells treated with MOI 100 and 1000, respectively).

LDH is an extremely stable cytoplasmic enzyme that exists in the cytoplasm of intact cells, and it is released extracellularly once the cell membrane is damaged 37. The amount of LDH released after bacterial infection was measured to investigate the cell integrity affected by VNP20009 and HCS1. The results showed that the VNP20009-treated cells significantly increased LDH release in MC38 and CT26 cells (Figure 1H; *p* = 0.0188 in MC38 cells and *p* = 0.0024 in CT26 cells). These results indicated that HCS1 was much less toxic than the VNP20009 strain *in vitro*, suggesting that HCS1 may reduce damage to normal tissue in tumors during bacterial cancer therapy *in vivo* and improve biosafety.

### Comparison of efficacy of HCS1 and VNP20009 in MC38 xenograft mice

To investigate the pathogenicity of the new HCS1 strain in mice, we injected the bacteria into the mice through the tail vein and calculated the half-lethal dose (LD_50_) using the Reed-Muench method 38. Our results showed that the LD_50_ of HCS1 was 4.72×10^8^ CFU, which is about 72-fold higher than that of VNP20009 (Table S3).

**Figure 2:**
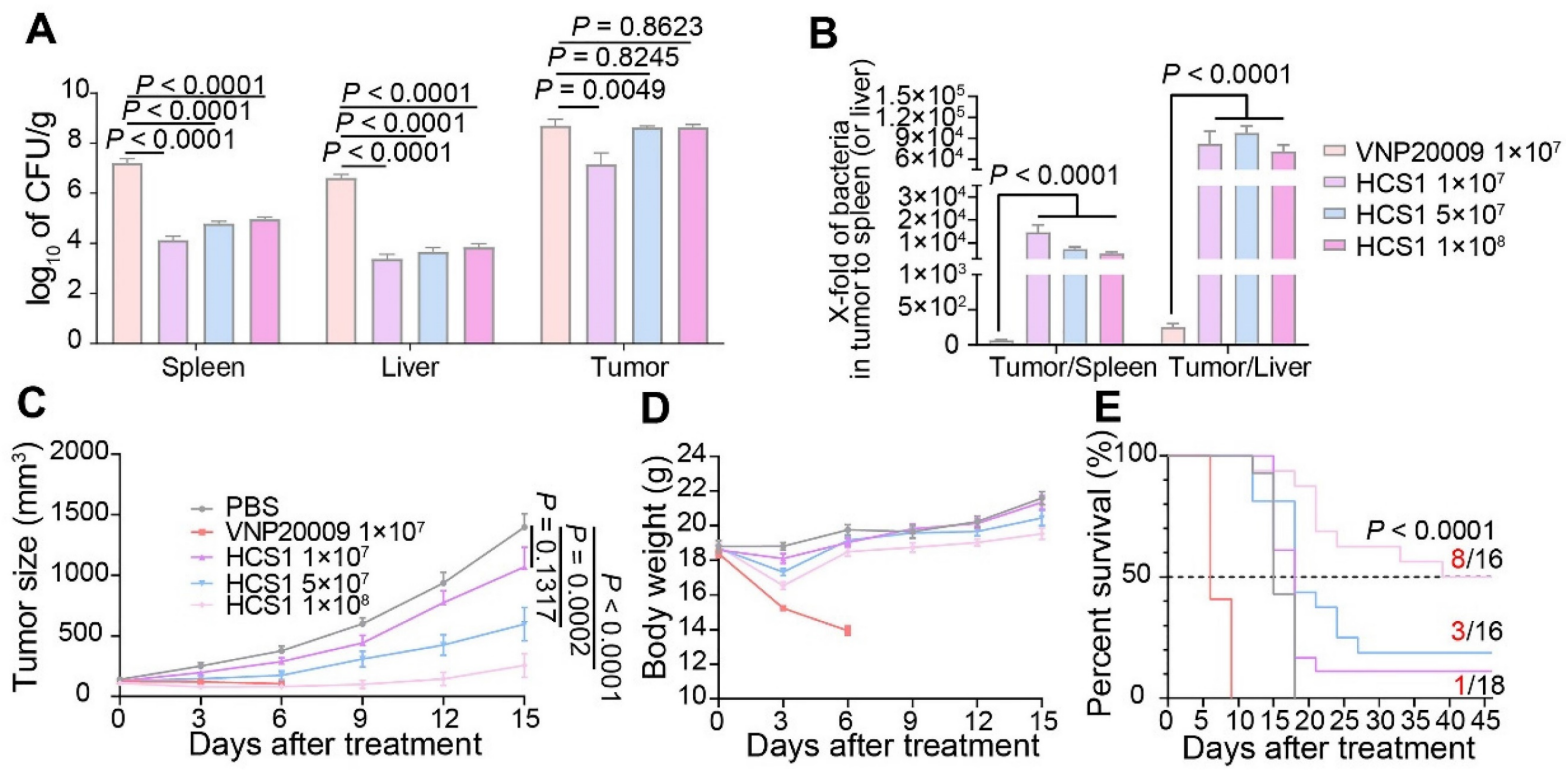
** Antitumor effects of VNP20009 and HCS1 strains in tumor-bearing mice.** (A) Bacterial distribution in MC38-bearing mice 3 days post bacterial injection (VNP20009 1×10^7^-treated group, n = 18; HCS1 1×10^7^-treated group, n = 13; HCS1 5×10^7^-treated group, n = 13; HCS1 1×10^8^-treated group, n = 10). (B) Fold changes of bacterial numbers in tumor tissue relative to bacterial numbers in the spleen or liver post bacterial infection. (C) Tumor growth curve in MC38-bearing mice. (D) Changes in body weight of mice after bacterial injection. (E) Animal survival curve of control and HCS1- and VNP20009-injected groups (PBS group, n = 14; VNP20009 1×10^7^-treated group, n = 22; HCS1 1×10^7^-treated group, n = 18; HCS1 5×10^7^-treated group, n = 16; HCS1 1×10^8^-treated group, n = 16). Data are presented as mean ± SEM.

Engineered *Salmonella typhimurium* has potent anticancer activity due to its good tumor-specific targeting and proliferation abilities 39,40. To explore the *in vivo* anticancer ability of HCS1, we established the MC38 mouse colorectal cancer xenograft model and administered it via the tail vein. Initially, we set the bacterial injection dose to 1×10^7^ based on previous studies 27; however, we found that HCS1 did not produce a decent tumor-inhibitory effect at this dose. Therefore, on the basis of LD_50_ results, we increased the dose of HCS1 and studied its distribution *in vivo*. The results showed that at the equal dose of 1×10^7^ CFU, the number of VNP20009 colonized in the liver or spleen was about 1000-fold higher than that of HCS1, even though the bacteria in tumor tissues also higher than that of HCS1-treated group. When the HCS1 was increased to 1×10^8^ CFU, we observed obviously increased bacterial colonization in tumor tissues without any notable increment in normal tissues (Figure 2A). Furthermore, the ratio of bacterial colonization in the tumor tissue to the liver or spleen was 100- to 1000-fold higher in the HCS1 injection group than that of the VNP20009 treatment group. (Figure 2B, *p* < 0.0001). At 3, 7 dpi, the bacterial distribution *in vivo* showed a similar tendency (Figure S2A-B). Higher doses of HCS1 bacteria resulted excellent tumor suppression (Figure 2C), among which tumor eradication was observed in 50% of the mice (8/16) treated with 1×10^8^ HCS1, while none of mice cured in the 1×10^7^ or 5×10^6^ CFU VNP20009-treated mice (Figure S3). When the mice were treated with 5×10^6^ VNP20009, the tumors suppression was comparable with 1×10^8^ HCS1, but the durable weight loss was observed in 5×10^6^ VNP20009-treated mice (nearly 20%, Figure S4). Furthermore, the results revealed a significant reduction in the bacterial colonization in the liver and spleen of mice treated with different doses of HCS1, as compared with those treated with VNP20009. Additionally, after the HCS1 injection, the weight of mice rapidly returned to normal (Figure 2D), and the survival time was prolonged (Figure 2E), especially in the 1×10^8^ HCS1-treated group, which was significantly improved compared with that of the VNP20009 treatment group. Meanwhile, to noninvasively monitor bacterial distribution *in vivo*, the light-emitting bacteria VNP20009^lux^ and HCS1^lux^ were injected into MC38 tumor-bearing mice intravenously, and bright signals in 1×10^8^ HCS1^lux^-treated mice were detected (Figure S5). We also established a BALB/c mouse bearing CT26 cancer to study the *in vivo* antitumor activity. We found that both HCS1 and VNP20009 treatments could induce robust tumor suppression. However, the high dose HCS1-treated group exhibited less loss of body weight compared with the VNP20009 treatment group (Figure S6). These results show that HCS1 can induce a substantial antitumor effect *in vivo*, while concurrently and remarkably reducing the toxicity to the hosts.

Cytolysin A (ClyA) is a bacterial toxin derived from *S. typhimurium* that kills cancer and cancer stromal cells via pore-forming activity 41. Attenuated *S. typhimurium* engineered has been used to express ClyA as a targeted therapy for pancreatic cancer in mice 31. Thus, we constructed the VNP20009^ClyA^ and HCS1^ClyA^ strains and validated high levels of ClyA protein expression in both strains via Western blot analysis following L-arabinose induction. No ClyA protein was detected in the absence of L-arabinose. Using the aforementioned studies as a guide, we selected a moderately therapeutic dose of HCS1 combined with ClyA for treatment. Our results showed that treatment with 5×10^7^ HCS1^ClyA^ significantly inhibited tumor growth and prolonged animal survival. Particularly, 40% of the mice (2/5) had complete tumor eradication (Figure S7). These findings substantiate the high drug-delivery potential of attenuated *Salmonella* HCS1 and its ability to effectively inhibit tumor growth.

### Immune activation of attenuated *Salmonella* HCS1

**Figure 3:**
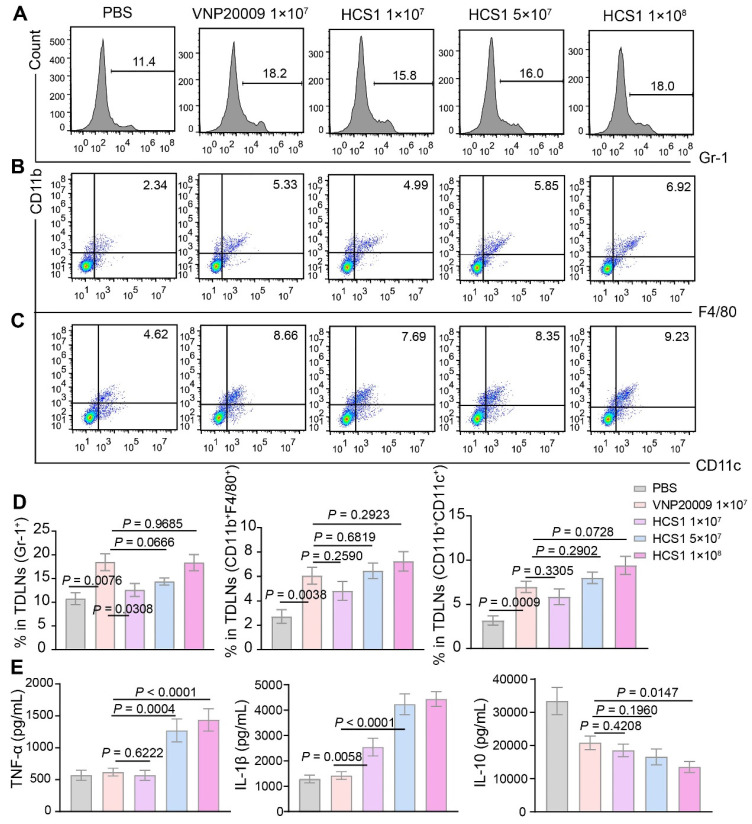
** Immune activation of bacteria in tumor-bearing mice.** (A to C) Immune cells in the TdLNs were collected post treatments for flow cytometry assessment after staining with Gr-1, CD11b, F4/80, and CD11c, and the corresponding flow cytometry quantitative analysis was performed (n = 4 mice per group). Gr-1: neutrophil, CD11b^+^F4/80^+^: macrophage, CD11b^+^CD11c^+^: dendritic cell. (D) Quantitative analysis of immune cells in the TdLNs. (E) TNF-α, IL-1β, and IL-10 levels in tumors after various treatments were analyzed using ELISA kits (PBS group, n = 14; VNP20009 1×10^7^-treated group, n = 19; HCS1 1×10^7^-treated group, n = 20; HCS1 5×10^7^-treated group, n = 13; HCS1 1×10^8^-treated group, n = 14). Data are presented as mean ± SEM. TdLNs: tumor-draining lymph nodes.

Bacterial treatment can recruit abundant immune cells, such as neutrophils, macrophages, and dendritic cells, into the tumor microenvironment 42. Therefore, we assessed immune cell infiltration by neutrophils, macrophages, and dendritic cells. On day 3 after bacterial injection, we collected tumor-draining lymph nodes of mice for flow cytometry analysis. Results showed that the number of neutrophils and macrophages in the bacterial injection group increased compared with that in the PBS group. Further exploration revealed that conversion of M2-like macrophages into M1-like macrophages upon bacterial treatments, we saw this effect was further enhanced in 1×10^8^ CFU HCS1-treated mice (Figure S8). No significant difference was present between the high dose HCS1 and VNP20009 treatment groups (Figure 3A-B, and 3D). Meanwhile, the number of dendritic cells exhibited a similar change (Figure 3C-D), indicating that the effectiveness of the antigen-presenting process increased. However, no significant population change was notified with CD4^+^ and CD8^+^ T cells (Figure S9).

Cytokine production plays an essential role in maintaining immune balance and regulating pathological processes such as immune diseases and cancer 43,44. We measured the concentrations of inflammatory cytokines in tumor tissue 3 days after bacterial treatment and observed that the levels of inflammatory cytokines (TNF-α and IL-1β) were increased in the bacterial treatment groups. The inflammatory cytokine levels in the high dose HCS1 treatment group were significantly enhanced (Figure 3E; *p* < 0.0001 for TNF-α and IL-1β). This result was consistent with the *in vitro* tests when bacteria were co-incubated with RAW264.7 at MOI 100 for 6 and 12 h and the cytokine contents and LDH release in the supernatant was determined, demonstrating that HCS1 did increase the release of antitumor inflammatory cytokines such as TNF-α and IL-1β (Figure S10). Additionally, the levels of the anti-inflammatory cytokine IL-10 were decreased in the bacteria-treated group, and the levels in the high dose HCS1 treatment group were significantly lower than those in the VNP20009 treatment group (Figure 3E; *p* = 0.0147). Even on day 7 after bacterial treatment, the inflammatory cytokines manifested similar changes (Figure S11). These results indicate that attenuated *Salmonella* HCS1, especially with high dose treatment, has excellent immune activation and good safety profiles.

### Anticancer mechanism of attenuated *Salmonella*

**Figure 4:**
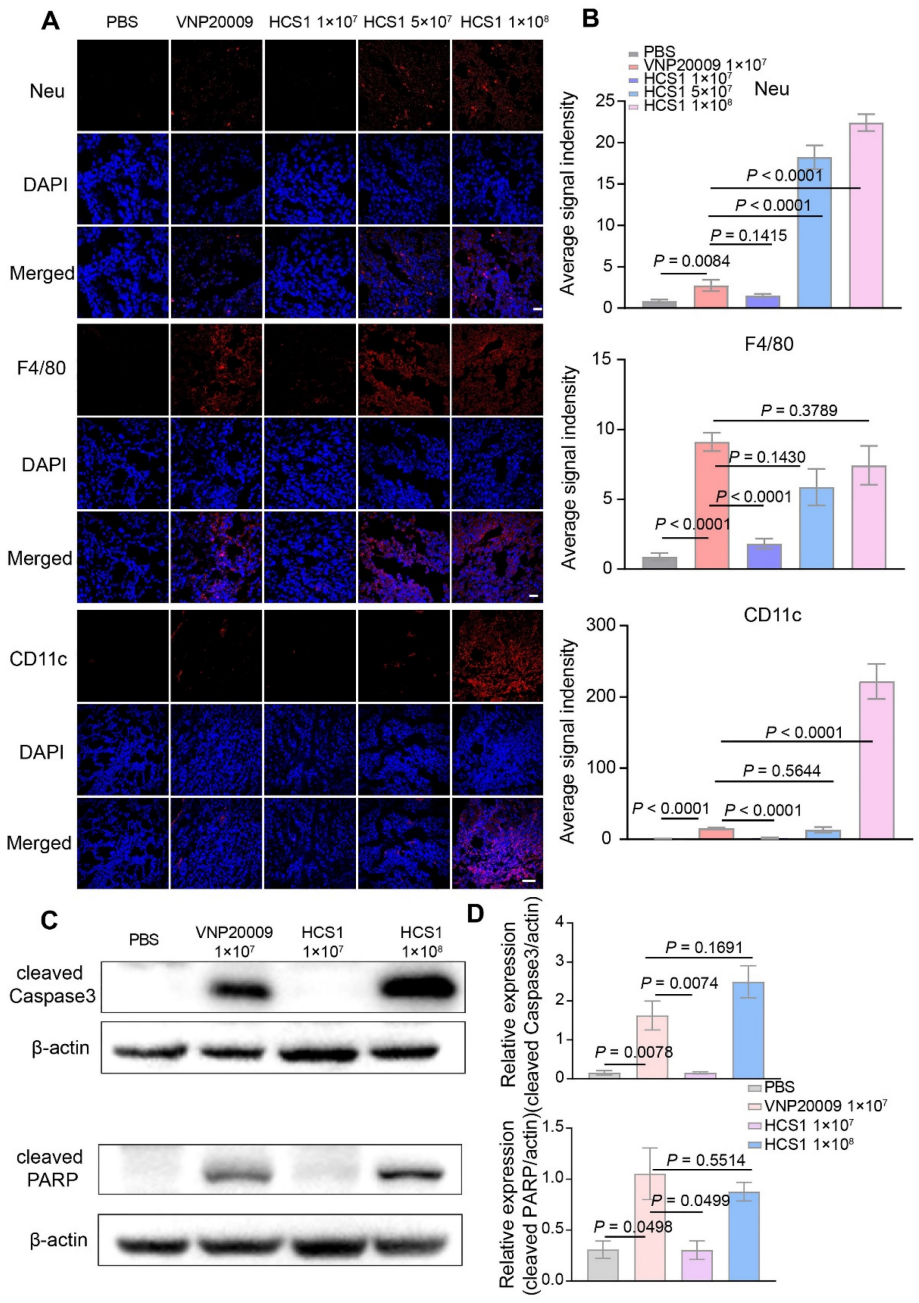
** Immune cell infiltration in the TMEs and cell apoptosis after bacterial treatment.** (A) Immunofluorescence was performed on tumor tissues to analyze the infiltration of neutrophils (Neu), macrophages (F4/80), and dendritic cells (CD11c). Nuclei were stained with DAPI. The scale bar is 20 μm for neutrophils and macrophages, and 50 μm for dendritic cells, n = 3). (B) Quantitative analysis of average fluorescent signals in the tumor. (C) Expression of cleaved Caspase3 and PARP protein in the tumor at 1 dpi after bacterial treatment was determined using Western blot (n = 3). (D) Quantitative expression of cleaved Caspase3 and PARP protein.

To further explore the anticancer mechanism of HCS1, we sectioned tumors for immunofluorescence staining 3 days post bacterial injection. Our results showed an increase in the neutrophils and dendritic cells in the high dose HCS1 treatment group, and the macrophages in the tumor was equivalent to that observed in the VNP200009 treatment group (Figure 4A-B). These results demonstrated that the attenuated *Salmonella* HCS1 could enhance immune activation *in vivo*.

*Salmonella* can induce the upregulation of tumor cell apoptosis, thereby inhibiting the proliferation of tumor cells 45,46. Caspase3, a cysteine-aspartic acid protease, is a key zymogen in cell apoptosis that is activated upon cleavage by initiator caspases during apoptotic flux 47. PARP undergoes protein post-translational modification enzyme activity and is present in most eukaryotic cells, specifically recognizes and binds to the broken end of DNA. PARP is the primary cleavage target of Caspase3 *in vivo* during the early stages of apoptosis, leading to loss of DNA repairing. Caspase3 can hydrolyze 116 kDa PARP into an 89 kDa fragment. PARP cleavage is considered as an important indicator of apoptosis and is generally used to indicate the Caspase3 activation 48,49. We collected tumor tissues on the first day after bacterial treatment, obtained protein samples through lysis, and performed Western blot to detect Caspase3 and PARP cleavages. The analysis found that after high dose HCS1 treatment, the proportion of cleaved Caspase3 and PARP was significantly higher than that in the PBS treatment group and comparable to that in the VNP20009 treatment group (Figure 4C-D,* p* = 0.1691 for cleaved Caspase3 and *p* = 0.5514 for cleaved PARP between the VNP20009-treated and 1×10^8^ CFU HCS1-treated groups). The TUNEL assay and Ki67 staining also indicated cell apoptosis was induced along with inhibited cell proliferation by bacterial treatment (Figure S12). These findings can explain why the better antitumor activity was achieved by the high dose of HCS1.

### Biosafety analysis of HCS1 and VNP20009

**Figure 5:**
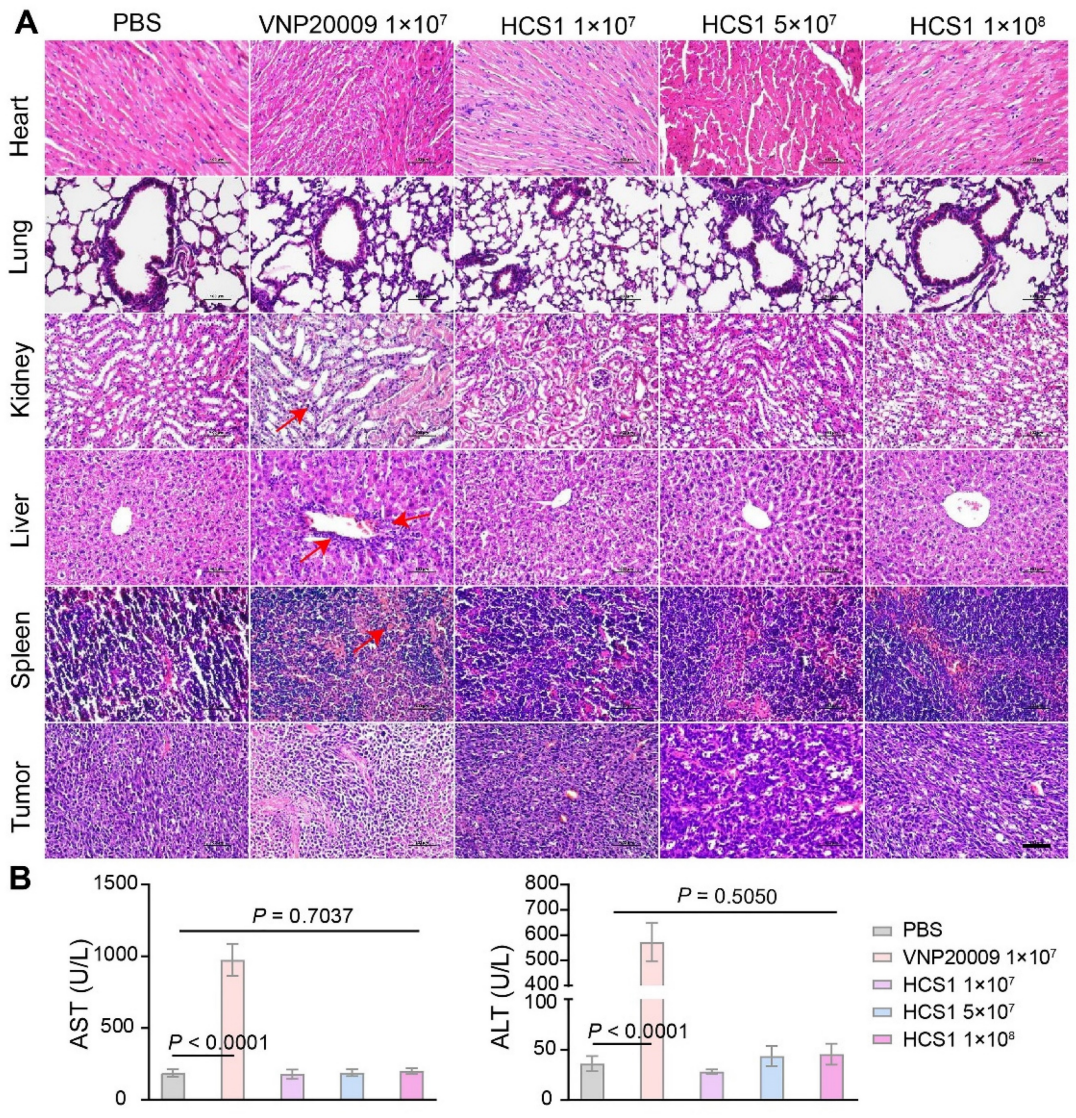
** Biosafety assessment of VNP20009 and HCS1.** (A) H&E staining was performed on organs (heart, liver, spleen, lung, and kidney) and tumor tissues 7 days post bacterial injection (n = 3, scale bar = 100 μm). (B) AST and ALT levels in the serum were analyzed 7 days post bacterial injection (n = 9).

Bacterial infections typically trigger systemic inflammatory responses, which can cause damage to the host. Here, the organs and tumors of mice from different groups were isolated and subjected to H&E staining to investigate the biosafety of the engineered strain HCS1. As shown in Figure 5A, H&E staining revealed no significant difference between the heart and lung in the bacterial treatment and control groups. However, liver, spleen, and kidney injuries were more severe in the VNP20009-treated group than in the HCS1-treated groups, even in the high dose HCS1-injected group. Additionally, H&E staining reflected stronger tumor cell destruction in the high dose HCS1 treatment (Figure 5A). Immunofluorescence analysis also revealed more immune cell infiltration in liver and spleen upon VNP20009 treatment, which suggests more serious inflammation triggered in these organs (Figures S13 and S14). As liver damage can be used as an indicator to evaluate biosafety, we checked clinical chemistry parameters, such as ALT and AST levels, in serum to further study the biosafety. The results showed that serum ALT and AST levels were significantly higher in the VNP20009 treatment group than in the HCS1 treatment groups (Figure 5B, *p* < 0.0001), which were comparable to those in the control group. In addition, the content of PLT and RBC of blood in VNP20009-treated mice was significantly reduced with whole blood analysis (Figure S15). These findings suggest that attenuated *Salmonella* HCS1 may not cause notable toxicity *in vivo* and exert high biosafety when used for cancer immunotherapy.

### Gene expression analysis after VNP20009 and HCS1 treatment

**Figure 6:**
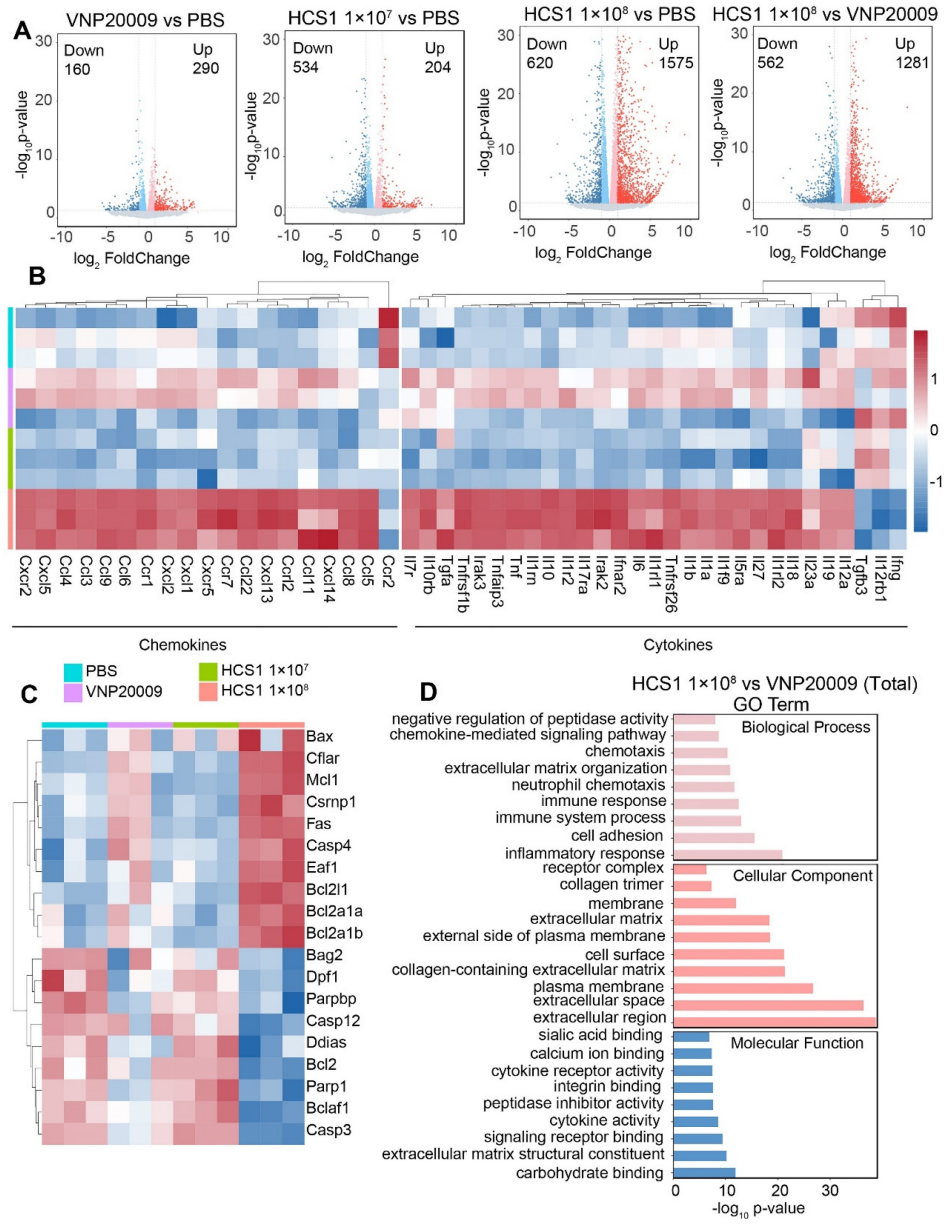
** Gene expression analysis in the TMEs using RNA sequencing.** (A) Volcano plot analysis of differentially expressed genes in the TMEs of mice treated with PBS, VNP20009, or different doses of HCS1. Log2 (fold change) is shown on the x-axis and the ‑log10 (p-value) on the y-axis. The upregulated genes, downregulated genes, and genes without significant diversity are presented in red, blue, and gray, respectively (n = 3 mice per group). (B) Heat maps of selected gene panels involved in chemokines and cytokines (n = 3 mice per group). (C) Heatmap of genes involved in cell apoptosis (n = 3 mice per group). Upregulated and downregulated genes are presented in red and blue, respectively. (D) GO enrichment analysis of biological functions associated with differentially expressed genes.

To investigate the effect of VNP20009 and HCS1 treatment on gene expression, we performed RNA sequencing on tumor tissues, the quality statistics of sequenced samples are displayed in Table S4. We found that a higher number of genes were upregulated in the HCS1-treated group than in the PBS- and VNP20009-treated groups (Figure 6A). Furthermore, functional annotation revealed that several immune activation-related genes were upregulated in the high dose HCS1-treated group, including genes involved in the inflammatory response, chemotaxis (Figure 6B), and immune response (Figure S16). These results were consistent with cytokine measurements presented in Figure 3E. However, the IL-10 levels in RNA sequencing were inconsistent with those in the ELISA experiments, indicating differences between mRNA and protein levels. The expression of apoptosis-related genes was analyzed (Figure 6C), and the results were consistent with Western blot results, showing an increase in the levels of cleaved Caspase3 and PARP (Figure 4C). Moreover, Caspase3 and PARP levels were reduced after high dose HCS1 treatment. GO analysis was conducted to further explore differential genes in terms of biological function. The results revealed the top enriched GO terms and were grouped into three functional categories: biological process, cellular component, and molecular function (Figure 6D and Figure S17).

## Discussion

In this study, we aimed to develop a new strain of *S. typhimurium* to accelerate the clinical translation of oncolytic bacteria with high safety and strong anticancer activity. A newly attenuated strain, HCS1, was developed based on VNP20009 by knocking out *relA* and *spoT*. HCS1 exhibited comparable in vitro growth to VNP20009 but significantly lower cytotoxicity and invasiveness In the MC38 tumor-bearing mouse model, HCS1 induced abundant immune cell infiltration and released high levels of antitumor inflammatory cytokines TNF-α and IL-1β and anti-inflammatory factor IL-10. Compared with VNP20009, HCS1 maintained good safety profiles and excellent antitumor effects at high doses *in vivo*.

BMCT has become prominent owing to its higher tumor-targeting specificity, tissue penetration, and lower toxicity compared with traditional tumor therapeutic options, such as surgical resection, radiotherapy, and chemotherapy. *Salmonella* is widely used as an effective strain 50, and VNP20009 has good antitumor activity in certain tumor types and has entered clinical trials. However, its efficacy needs further improvement 13,51. Poor clinical outcomes with VNP20009 may be due to insufficient bacterial colonization and substantially adverse effects at high doses 21. Therefore, constructing mutant strains of *S. typhimurium* with high tumor-targeting specificity, deep tissue penetration, and low systemic toxicity is necessary. The ΔppGpp strain obtained by knocking out the virulence-related genes *relA* and *spoT* in the wild-type *S. typhimurium* 14028s showed good targeting and low toxicity 52,53. Based on this finding, we knocked out these two genes in VNP20009 and created the HCS1 strain that exhibited improved safety profiles and demonstrated robust anticancer efficacy and low systemic toxicity even at 10-fold higher doses. This makes HCS1 an excellent candidate for *S. typhimurium*-mediated tumor therapy and its clinical trials.

Although VNP20009 induce significant tumors shrinkage, the serious systemic toxicity cannot be ignorable. HCS1 exhibited low cytotoxicity and invasion ability. *In vivo* experiments revealed that HCS1 can promote the infiltration of immune cells into tumor tissues, such as neutrophils, macrophages, and dendritic cells, which further increase the secretion of pro-inflammatory cytokines, such as TNF-α and IL-1β. Additionally, the cytotoxicity of HCS1 to the liver and spleen was significantly reduced. Further, we found that high dose HCS1 treatment significantly increased cell apoptosis in tumor tissues, which can explain its robust therapeutic effect. Furthermore, RNA sequencing showed that compared with the VNP20009 treatment, HCS1 treatment upregulated the expression of massive immune response-related genes. Studies have shown that both VNP20009 and ΔppGpp can be used as ideal drug delivery carriers 18,50. Correspondingly, HCS1 also maintains good drug-loading properties and expression capabilities, making it an excellent drug-delivery vector. Hence, the engineered HCS1 allows administration of higher dose for sufficient delivery of target therapeutic payloads with minimized toxicity 27,54. Future studies should be carried out in more types of cancer models, including the clinically more relevant orthotopic cancer models, to verify the anticancer activities.

In conclusion, compared with VNP20009, the HCS1 strain has reduced cell invasion and cytotoxicity *in vitro*, with higher biosafety and better therapeutic effect in MC38 tumor-bearing mice with 50% of mice cured. Therefore, HCS1 may have greater clinical application potential than VNP20009 in terms of anticancer activity and biological safety, providing a new strategy for the clinical application of bacterial therapy.

## Supplementary Material

Supplementary figures and tables.
