# Supplementary material for: Engineered oncolytic bacteria HCS1 exerts high immune stimulation and safety profiles for cancer therapy
Source: Theranostics. 2023 Oct 16;13(15):5546–60. doi: 10.7150/thno.87340 (PMC10614684; doi:10.7150/thno.87340)
Supplement: Supplementary file 1 — Supplementary figures and tables. [file thnov13p5546s1.pdf]

Supplemental Materials for

Engineered oncolytic bacteria HCS1 exerts high immune stimulation and safety profiles for cancer therapy

Yujie Sun[1], Yanxia Guo[1], Xiaoqing Liu[1], Jinling Liu[1,2], Honglai Sun[1], Zhongying Li[1], Min Wen[3], Sheng-Nan Jiang[4], Wenzhi Tan[1,5], Jin Hai Zheng[1*]

[1]School of Biomedical Science, Hunan University, Changsha 410082, China.

[2]College of Biology, Hunan University, Changsha 410082, China.

[3]Department of Neurosurgery, Guangzhou First People's Hospital, Guangzhou 510180, China.

[4]Department of Nuclear Medicine, the Second Affiliated Hospital of Guangzhou Medical University, Guangzhou, 510260 China.

[5]School of Food Science and Bioengineering, Changsha University of Science & Technology, Changsha, Hunan 410114, China.

***Corresponding author.**
Email: jhzheng@hnu.edu.cn

Table S1. List of bacteria used in the study

Name	Feature	Source	Application
SM10 λpir	Kan^R, λpir^+	our lab	pDM4 host, conjugative strain
VNP20009		our lab	background strain
pDM4	Cm^R, $sacB^+$	our lab	suicide vector
pDM4-*relA*	Cm^R, $sacB^+$	this study	for *relA* deletion
pDM4-*spoT*	Cm^R, $sacB^+$	this study	for *spoT* deletion
Δ*relA*		this study	*relA* deletion strain
HCS1		this study	*relA* and *spoT* deletion strain

Table S2. List of primers used in the study

Primer name	Primer sequence (5′-3′)
relA-del-1	GGACGAGCTCAGCAACAGCATGTCAGCATC
relA-del-2	ATTACTGTCTGGGGTCGTCCTCTCCTTAAGGGACC
relA-del-3	CCTTAAGGAGAGGACGACCCCAGACAGTAATCATGT
relA-del-4	CCAGGGCCCATTCACCCAGTTCAGGTCGA
relA-F	ATGGTCGCGGTAAGAAGTGC
relA-R	TCAATCACATCCGGCACCTG
spoT-del-1	GGACGAGCTCCGATAATCACAGACGTAAGATACTCATGG
spoT-del-2	CGTTTTGGATTCATAGCGGGGCGACCCGCTTTGTGATTAACGACG
spoT-del-3	TCACAAAGCGGGTCGCCCCGCTATGAATCCAAAACGTTATGCGCG
spoT-del-4	CAGGGCCCTCAAACAGCAGGCGCTGCTGTTCATCTT
spoT-F	TTCAAACCTACCTGCCGGAAG
spoT-R	CTAGTTTCGGTTACGGGTGAC

Table S3. Measurement of the median lethal dose in mice post-bacterial infection

	Injection dose	Deaths /Total mice	LD_{50} (CFU)
VNP20009	1×10^6	0/16	6.54×10^6
	1×10^7	10/16	
HCS1	1×10^8	2/16	4.72×10^8
	1×10^9	15/16	

*LD_{50}: HCS1/VNP20009 = 72.

Table S4. Sequencing quality statistics of each RNA-Seq sample

Sample ID	Total number of sequenced reads	Total number of uniquely mapped reads*	RNA integrity number (RIN)	Ratio of all reads aligned to rRNA regions to total uniquely mapped reads (rRNA rate)	Ratio of exon-mapped reads to total uniquely mapped reads (Expression Profile Efficiency)	Total number of detected transcripts with reads≥1
P1	50419226	41987973	9.8	0.89	83.28	19625
P2	47495402	38631361	9.8	0.62	81.34	19464
P3	48388716	40178362	9.8	0.71	83.03	19622
VNP-1	49860556	41737866	8.5	0.65	83.71	19701
VNP-2	48489858	40133970	6.4	0.67	82.77	19610
VNP-3	49214616	41474735	9.4	0.64	84.27	19515
VP1-1-1	48386324	40302889	9.4	0.68	83.29	19372
VP1-1-2	49523512	41232686	9.6	0.57	83.26	19683
VP1-1-3	50269952	41622339	9.2	0.63	82.8	19610
VP1-10-1	48102788	39869394	4.9	0.69	82.88	19709
VP1-10-2	49461616	41288038	6.5	0.65	83.47	19582
VP1-10-3	49053932	39523919	6.0	1.2	80.57	19979

*GRCm39 was used as reference genome.

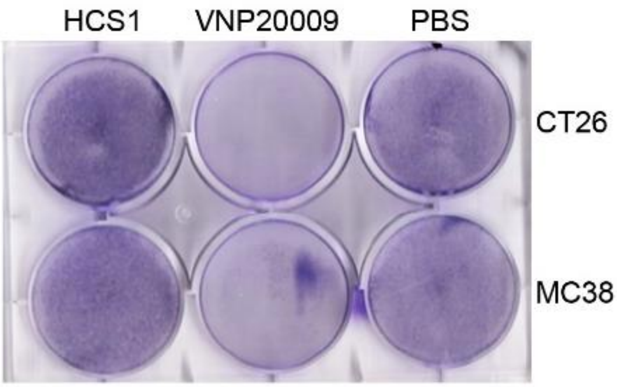

Figure S1. Viability of cells assessed through crystal violet staining. MC38 and CT26 cells were co-cultured with HCS1 and VNP20009 at MOI = 1000 for 12 h. Subsequently, the bacteria were removed, and the culture medium was replaced with basic DMEM containing 150 μg/mL gentamycin for another 24 h of culture.

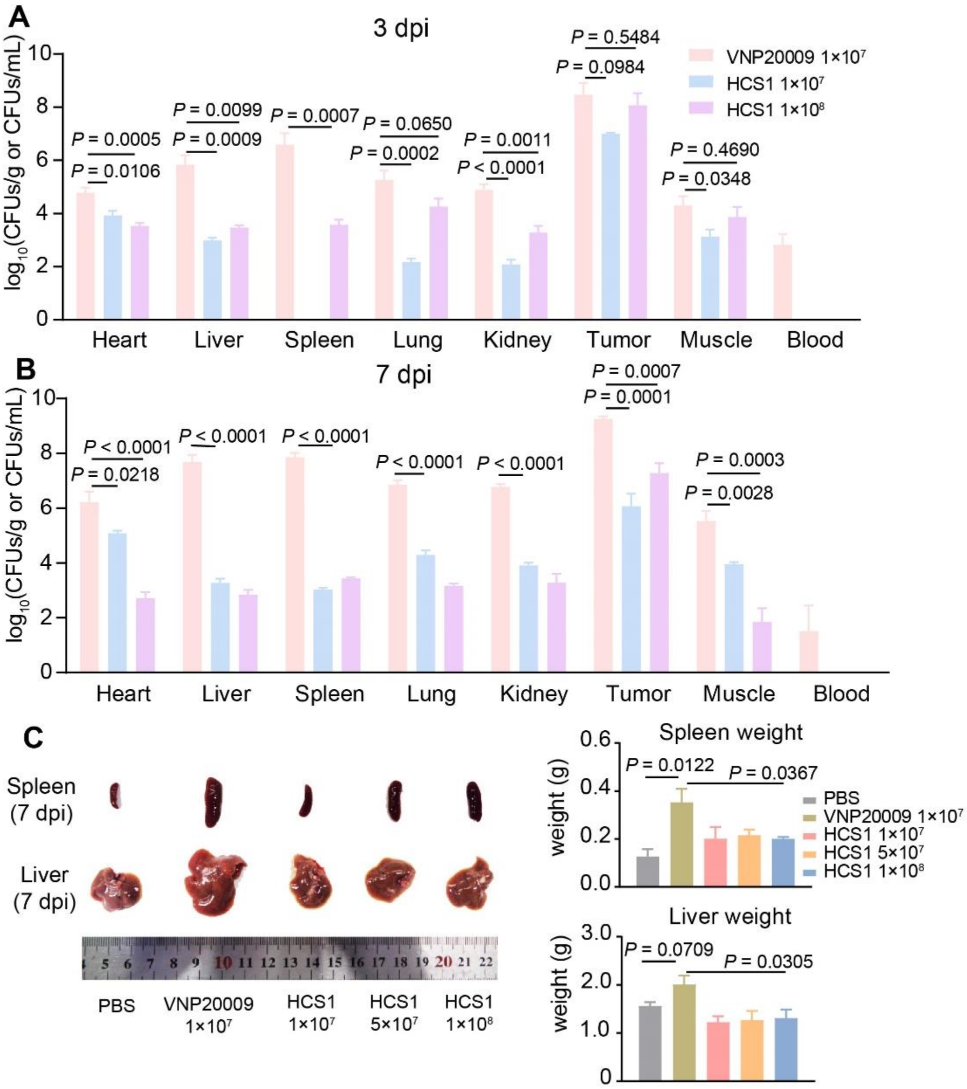

Figure S2. Bacterial distribution in MC38-bearing mice post injection. Bacterial viable counts in tumor-bearing mice at 3 dpi (A), 7 dpi (B) post bacterial infection. (C) Image and weight of spleen and liver at 7 dpi, 4 mice per group.

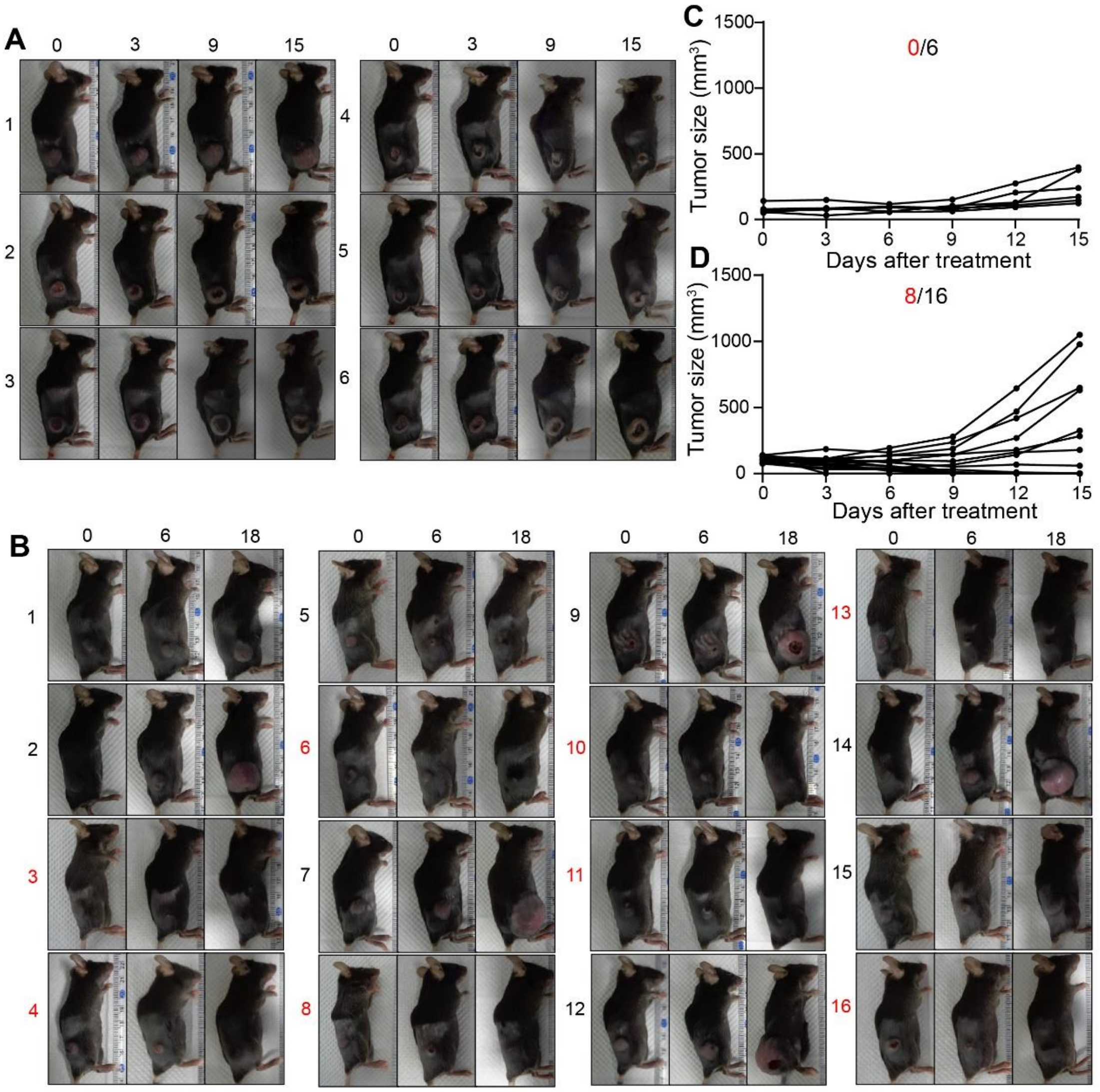

Figure S3. Mice photos and individual tumor growth curves. (A) Photographs of tumor-bearing mice treated with 5×10^{6} CFU VNP20009. (B) Mice treated with 1×10^{8} CFU HCS1, red numbers indicate mice with complete tumor eradication. (C) Tumor size changes from individual mice treated with 5×10^{6} CFU VNP20009. (D) Tumor changes from individual mice with 1×10^{8} CFU HCS1 treatment, 8 out of 16 mice showed complete tumor eradication.

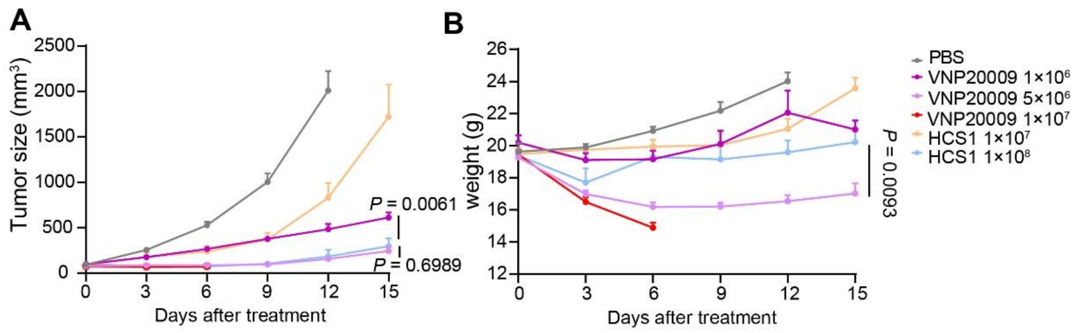

Figure S4. Tumor growth and body weight change in MC38 tumor-bearing mice. (A) Tumor growth curve post bacterial treatments in MC38-bearing mice. (B) Body weight change after bacterial treatments. Data are presented as mean ± SEM, with 6 mice per group.

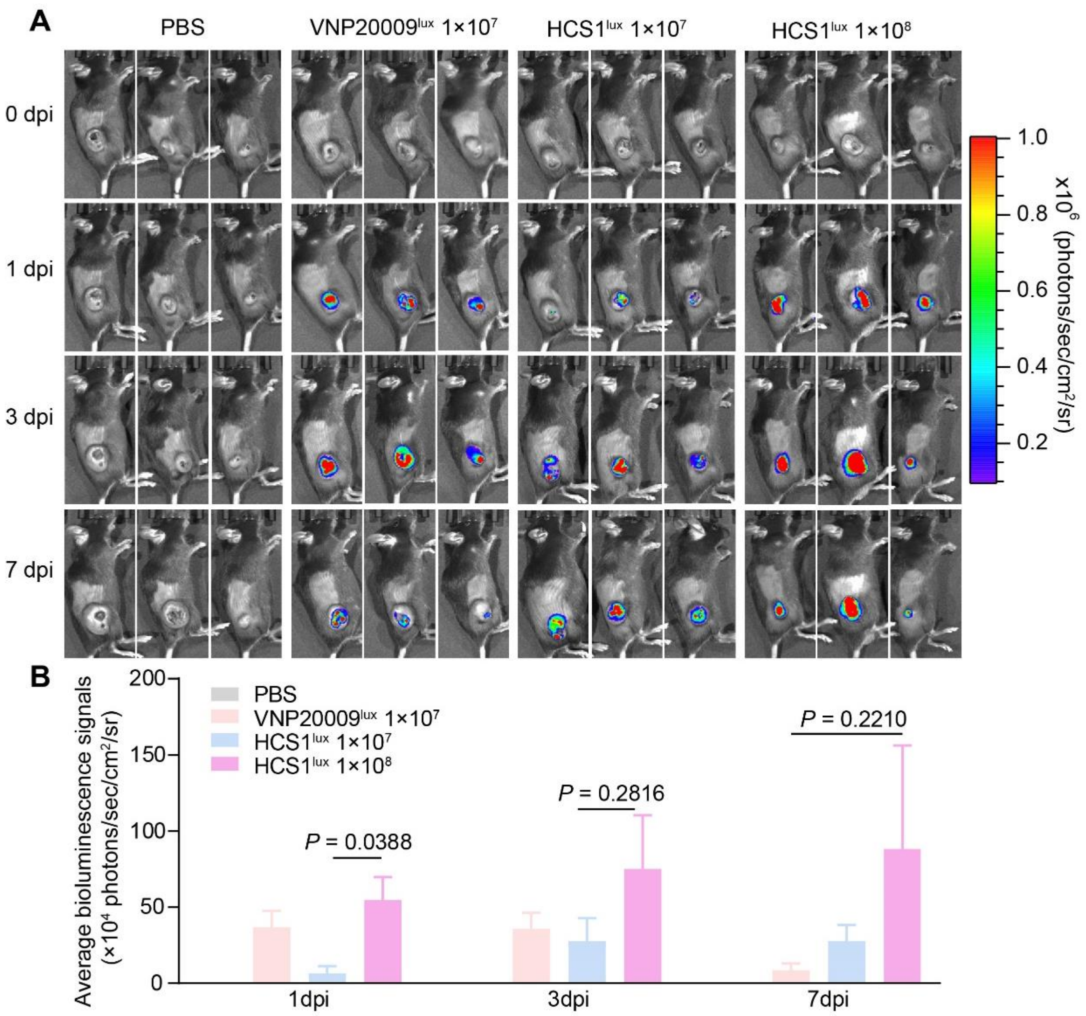

Figure S5. Noninvasive monitoring of bacterial distribution *in vivo.* (A) Bacterial bioluminescence detected by optical imaging. (B) Average bioluminescence intensity in tumors (n = 3 mice per group). Data are presented as mean ± SEM.

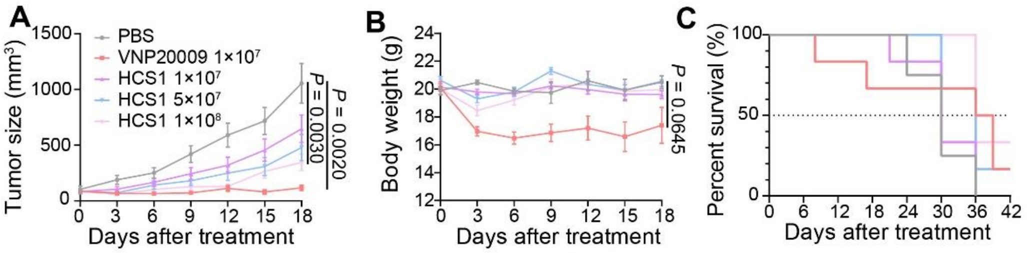

Figure S6. Antitumor effect of VNP20009 and HCS1 strains in the CT26 cancer model. (A) Tumor growth curve in CT26-bearing mice. (B) Change in mice body weights post bacterial injection. (C) Animal survival curve. Data are presented as mean ± SEM, 6 mice per group.

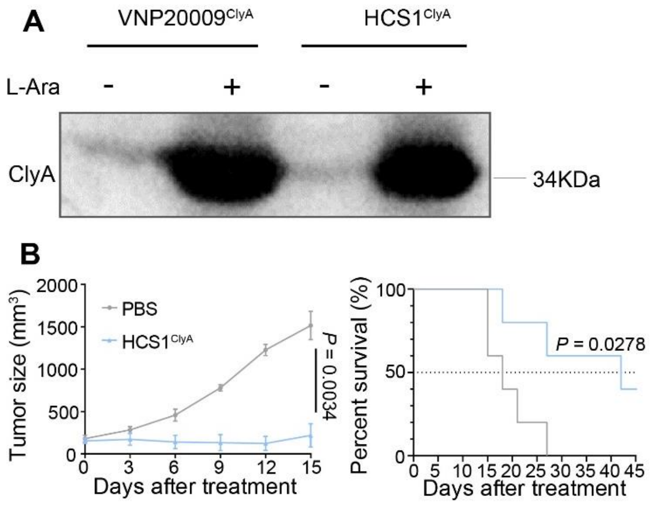

Figure S7. Application of HCS1 as a drug delivery vector. (A) Expression of ClyA in the bacterial culture was determined using Western blot with an anti-ClyA antibody without or with 0.2% L-arabinose induction *in vitro*. (B) Average tumor growth and mice survival in the MC38 cancer model after intratumoral injection of 5×10^{7} CFU of $HCS1^{ClyA}$.

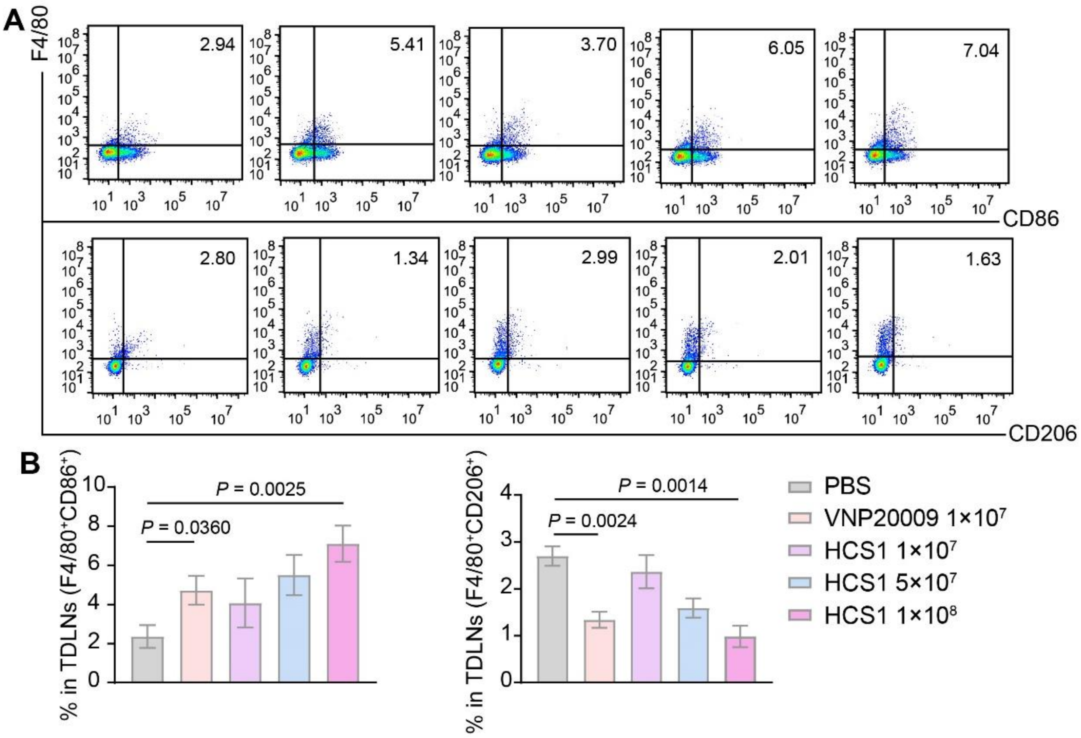

Figure S8. Flow cytometry analysis of macrophage polarization in TdLNs. (A) Samples were stained with F4/80 (macrophage marker) and CD206 (M2-like macrophage) or CD86 (M1-like macrophage) and analyzed by FACS. (B) Quantitative analysis of M1-like and M2-like macrophages. Data are presented as mean ± SEM, with 4 mice per group**.**

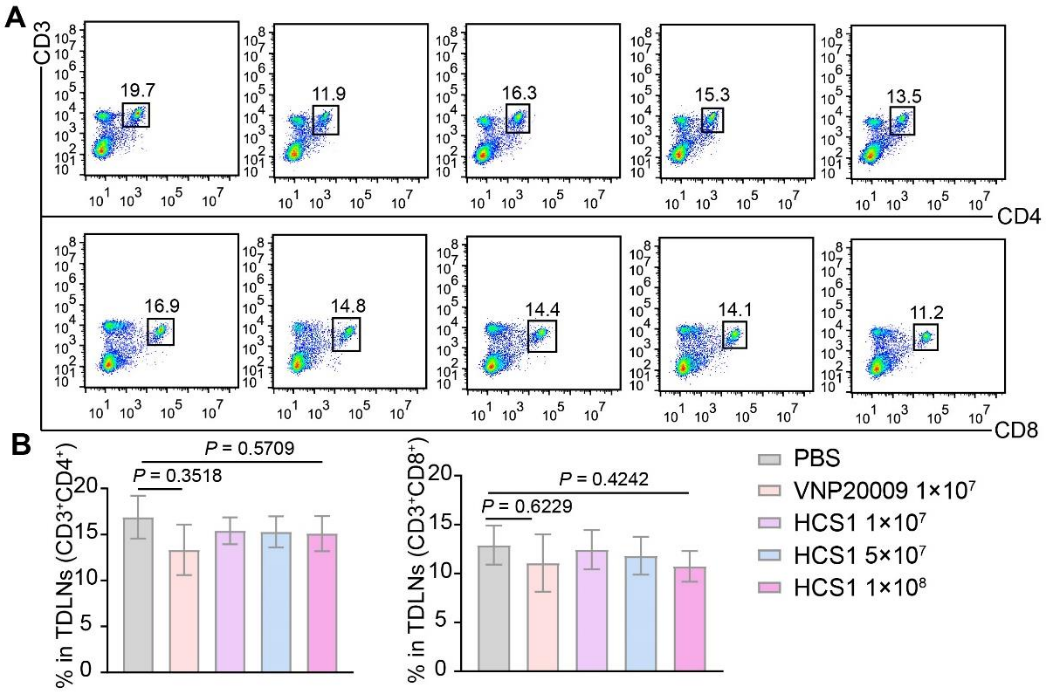

Figure S9. Flow cytometry analysis T cells in TdLNs. (A) Samples were stained with CD3 (T cell maker), and CD4 or CD8 and analyzed by FACS. (B) Quantitative analysis of CD4 and CD8 T cells. Data are presented as mean ± SEM, with 4 mice per group.

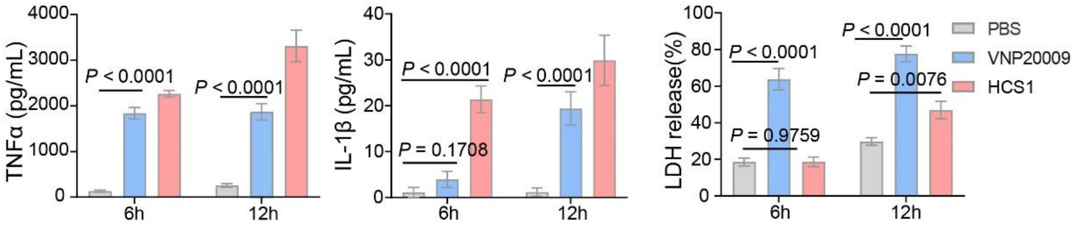

Figure S10. Detection of inflammatory cytokines *in vitro*. Bacteria were incubated with RAW264.7 at MOI 100 for 6 and 12 h, and TNF-α and IL-1β levels and LDH release in the supernatant were determined (n = 3). Data are presented as mean ± SEM.

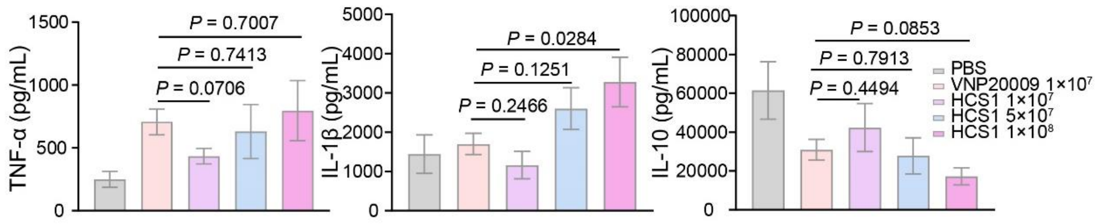

Figure S11. Inflammatory cytokine measurement in tumor tissues. TNF-α, IL-1β, and IL-10 levels in the tumors after various treatments were analyzed using ELISA kits 7 days post bacterial treatment (n = 5 mice per group). Data are presented as mean ± SEM.

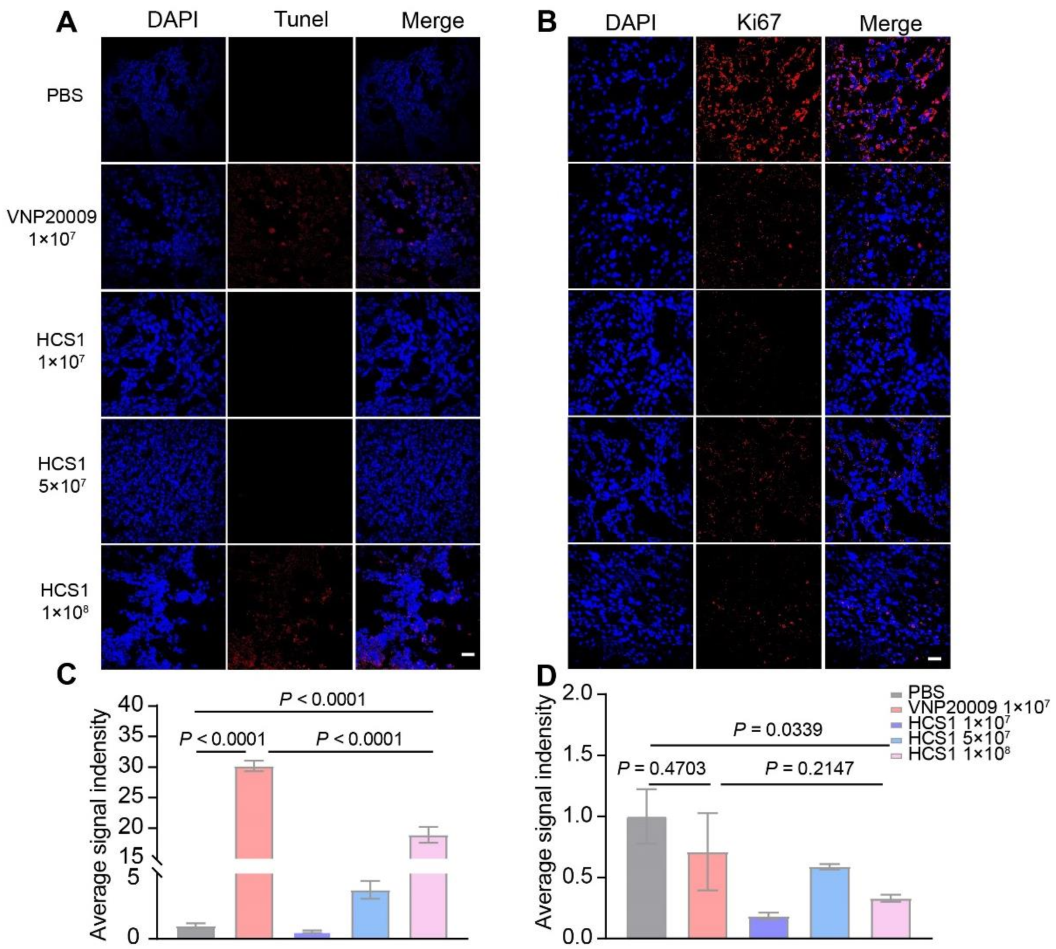

Figure S12. Immunofluorescence analysis of tumor tissues. Tumor sections from MC38-bearing mice at 7 dpi were stained with (A) TUNEL for cell apoptosis, and (B) Ki67 antibody for cell proliferation, nuclei were stained with DAPI (scare bar = 20 μm). Quantification of the fluorescence intensity of (C) TUNEL and (D) Ki67 normalized to the PBS control group, respectively. Data are presented as mean ± SEM, with 3 mice per group.

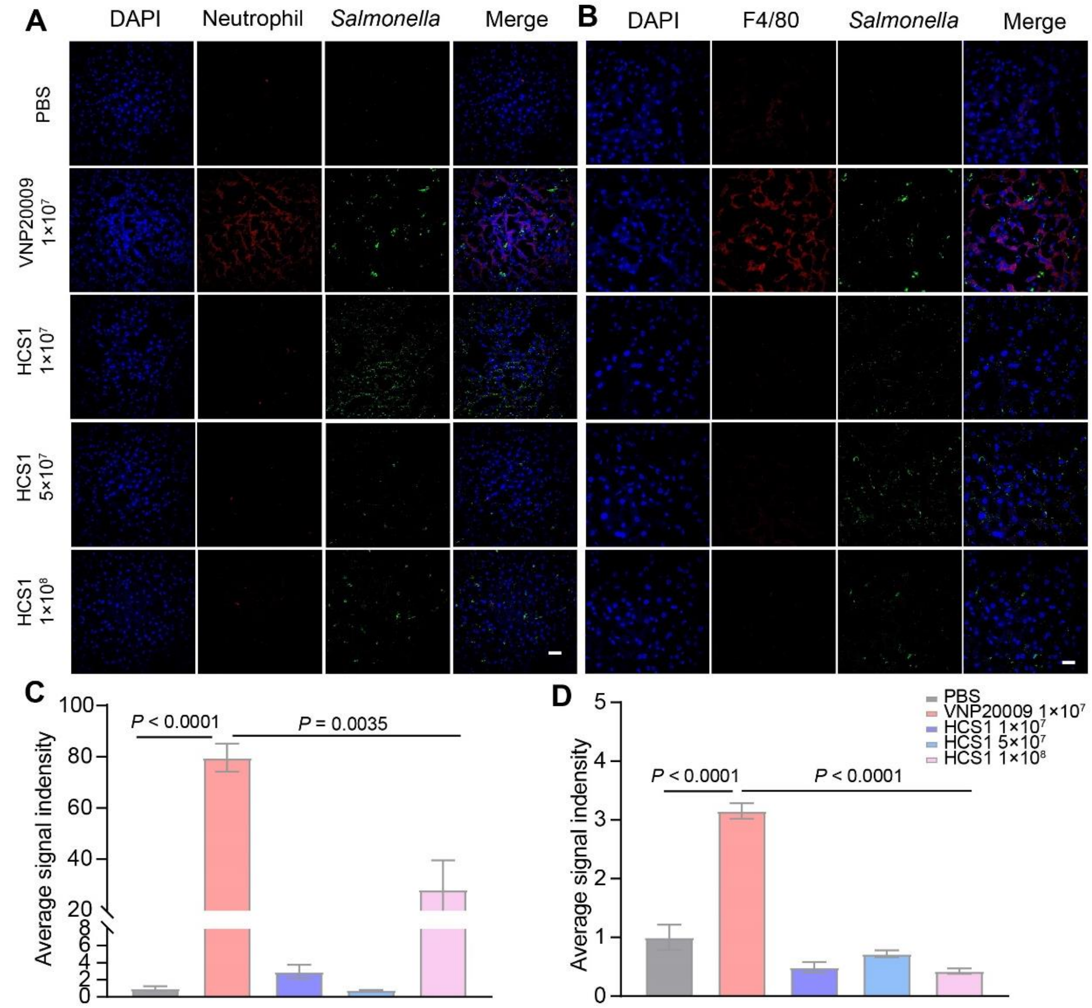

Figure S13. Detection of immune cell infiltration in liver tissues at 7 dpi. Immunofluorescence staining to check (A) neutrophils and (B) macrophages in contiguous liver slides. Quantification of the fluorescence intensity of (C) neutrophils and (D) macrophages normalized to the PBS control group, respectively. Data are presented as mean ± SEM, with 3 mice per group.

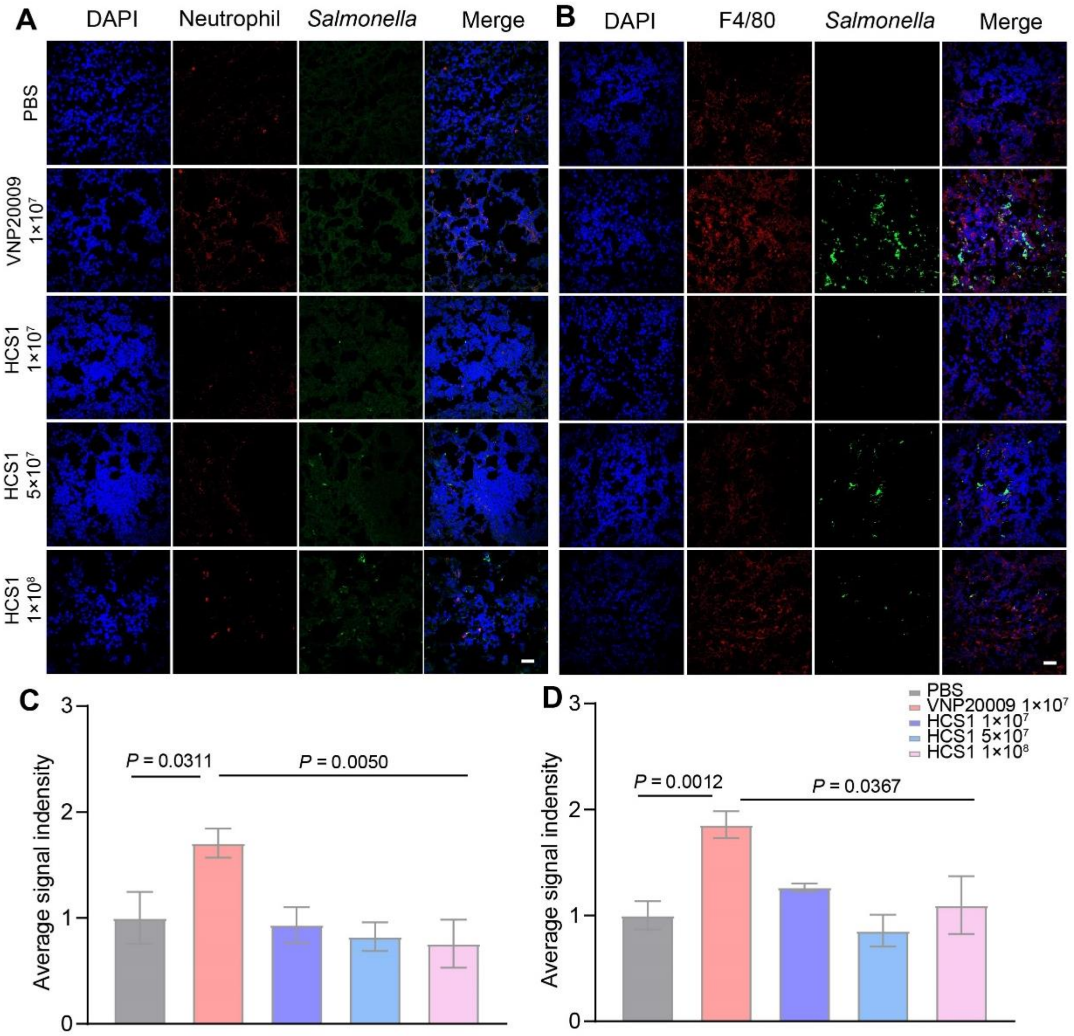

Figure S14. Detection of immune cell infiltration in spleen tissues at 7 dpi. Immunofluorescence staining to check (A) neutrophils and (B) macrophages in contiguous spleen slides. Quantification of the fluorescence intensity of (C) neutrophils and (D) macrophages normalized to the PBS control group, respectively. Data are presented as mean ± SEM, with 3 mice per group.

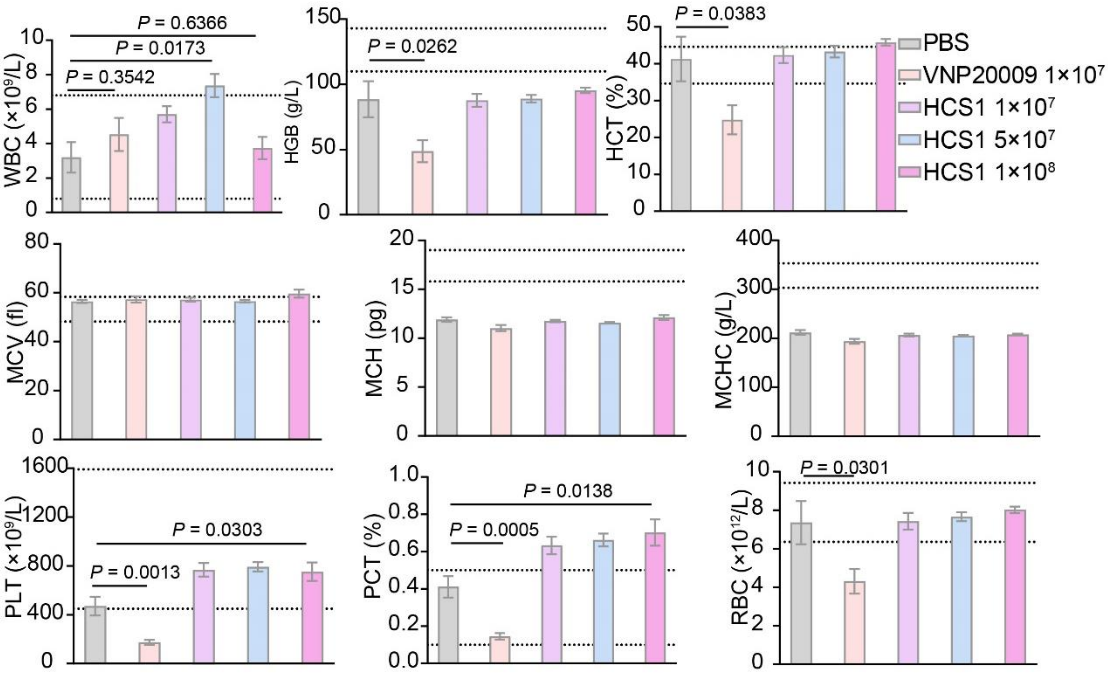

Figure S15. Whole blood analysis in mouse at 7 days post treatments. Whole blood was obtained from the mice via orbital blood collection and kept in EDTA-pretreated tubes, and then the samples were analyzed with a blood analyzer (Hemo 3600V, SHINOVA). Data are presented as mean ± SEM with 5-7 mice per group. WBC: White Blood Cell, HGB: Hemoglobin, HCT: Hematocrit, MCV: Mean Corpuscular Volume, MCH: Mean Corpuscular Hemoglobin, MCHC: Mean Corpuscular Hemoglobin Concentration, PLT: Platelet, PCT: Plateletcrit, RBC: Red Blood Cell.

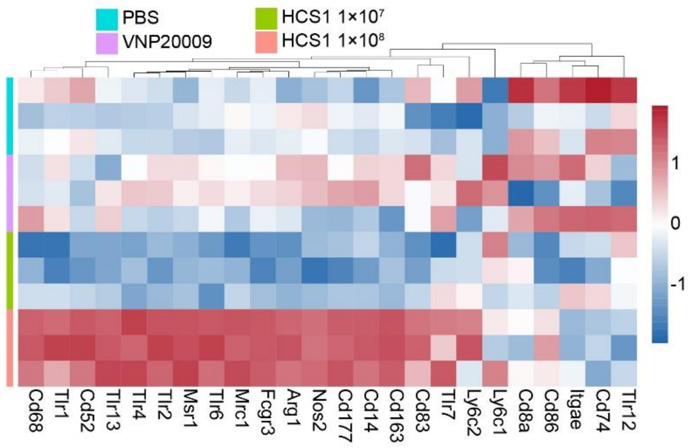

Figure S16. Heatmap of immune cell-related genes. Upregulated and downregulated genes are presented in red and blue, respectively (n = 3 mice per group).

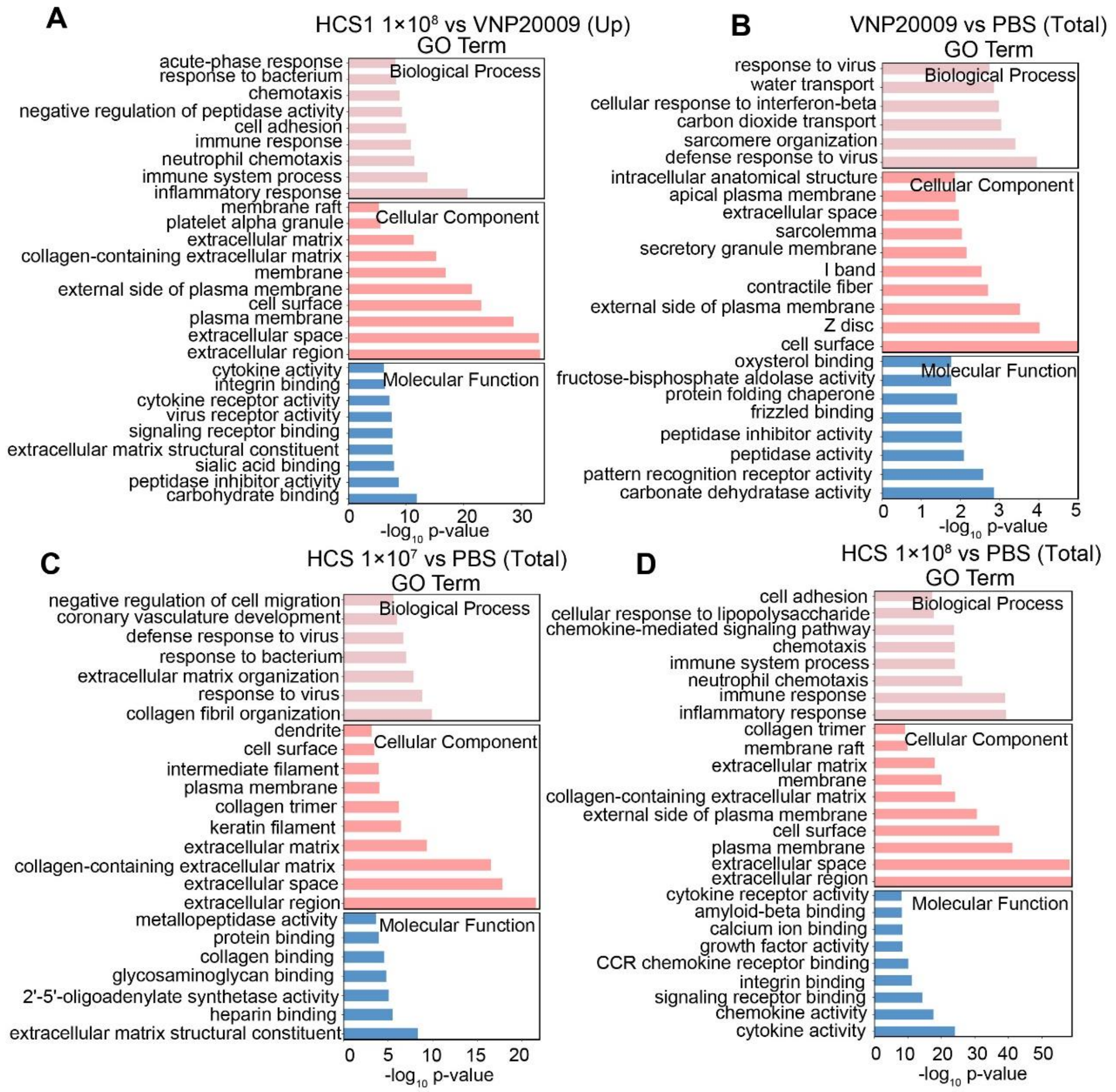

Figure S17. GO enrichment analysis of expressed genes. (A) Top enriched GO terms upregulated on HCS1 1×10^8 CFU treatment versus VNP20009 treatment. (B-D) Top enriched GO terms in VNP20009, HCS1 1×10^7 CFU, and HCS1 1×10^8 CFU treatment versus PBS treatment, respectively (n = 3 mice per group).
